# Catechin-Targeted Nano-Enhanced Colorimetric Sensor Array Based on Quantum Dots—Nano Porphyrin for Precise Analysis of Xihu Longjing from Adjacent Origins

**DOI:** 10.3390/foods14193360

**Published:** 2025-09-28

**Authors:** Yaqi Liu, Zhenli Cai, Yao Fan, Xingcai Wang, Meixia Wu, Haiyan Fu, Yuanbin She

**Affiliations:** 1 State Key Laboratory of Green Chemical Synthesis and Conversion, College of Chemical Engineering, Zhejiang University of Technology, Hangzhou 310032, China; kltslyq@163.com (Y.L.); czl21234@163.com (Z.C.); wangxingcai1994@126.com (X.W.); wmx@zjut.edu.cn (M.W.); 2 The Modernization Engineering Technology Research Center of Ethnic Minority Medicine of Hubei, School of Pharmaceutical Sciences, South-Central Minzu University, Wuhan 430074, China; fuhaiyan@mail.scueu.edu.cn

**Keywords:** Xihu Longjing identification, sensor array, quantum dots, nano porphyrin, nano-enhanced visual and fluorescent dual-mode

## Abstract

Aimed at addressing the increasingly serious problem of adulteration in Xihu Longjing, a catechin-targeted nano-enhanced visual and fluorescent dual-mode sensor array was constructed by nano porphyrins and quantum dots (QDs) for the precise analysis of Xihu Longjing from adjacent origins. This sensor array realizes the quantitative analysis of catechin enantiomers in Xihu Longjing through the selective combination of sensing units. It can accurately identify adjacent Xihu Longjing teas with different grades and storage times and can precisely detect samples with a low proportion of adulteration. At the same time, the flavor quality and antioxidant performance of Xihu Longjing tea can also be quantitatively evaluated. The dual-mode sensor array design proposed in this study provides a new idea for detecting minor differences in food authenticity and has significant application value for quality control in the tea industry.

## 1. Introduction

Longjing tea is one of the top ten famous teas in China [1]. With the surging popularity of Longjing tea worldwide, people have been experiencing an increasing demand for high-quality Longjing tea. Longjing tea not only has a rich taste and aroma but also boasts a variety of health benefits [2,3]. For example, Longjing can help remove free radicals in the body, reduce cell damage, and reduce the risk of chronic diseases such as cardiovascular and cerebrovascular diseases and cancer; increase metabolic rate; lower cholesterol; and so on [4,5]. Among which, Xihu Longjing, the Longjing teas originating from core production areas including Shifengshan, Meijiawu, Yunqi, and Hupao, owned the best reputation and the most outstanding quality. Nevertheless, different origins and processing technologies can induce variations in the quality of Longjing tea; even Xihu Longjing from adjacent origins can exhibit significant quality differences. For example, it is well known that the quality of Xihu Longjing produced in adjacent core origins was ranked as Shifengshan, Meijiawu, Yunqi, and Hupao, from highest to lowest, and their commercial value can differ significantly (by several to dozens of times in actual markets) even when the distance between different core production areas is as close as 3 km. Currently, there are many types of Longjing teas on the market, but their quality is uneven. The lack of strict grade classification standards leads to the phenomenon of shoddy or even false labeling occurring from time to time [6,7]. For conventional consumers, it is challenging to determine the authenticity of different Longjing teas solely based on sensory characteristics such as smell and appearance, due to the lack of effective on-site analysis methods [6,8]. Consequently, a simple and rapid detection method is urgently required to identify the authenticity and adulteration of Xihu Longjing from adjacent origins.

In recent years, many researchers have engaged in sensory evaluation [9], chromatography–mass spectrometry [10,11], spectroscopy [12,13], and other methods to analyze the authenticity of Longjing tea. However, for the sensory evaluation, it is difficult to form standards relying mainly on the professional experience and subjective judgment of researchers, which limits its wide application [14]. Chromatography–mass spectrometry is widely used for quality analysis of Longjing tea because of its strong separation ability and rapid analysis of various compounds simultaneously. Yin et al. used headspace solid-phase microextraction and gas chromatography–mass spectrometry to analyze the volatile components of Hunan black tea qualitatively. A total of 88 compounds were successfully extracted and determined from Hunan black tea. In addition, aroma-active compounds were identified by odor activity value (OAV) [15,16,17,18]. This method exhibits good sensitivity and selectivity. However, it requires complex pretreatment, expensive professional equipment, and specialized operation. Other than chromatography–mass spectrometry, spectroscopy is also widely used due to its properties, such as being simple to operate and rapid detection [19]. Li et al. used visible light and near-infrared spectroscopy to rapidly and simultaneously determine six primary fat-soluble pigments in green tea. Five kinds of three grades of tea samples were collected for spectral scanning and color determination, and the content of fat-soluble pigment was determined by high-performance liquid chromatography. Finally, the quantitative determination models of 6 pigments were established based on multiple linear regression of characteristic wavelengths, and their (2) were 0.975, 0.973, 0.993, 0.919, 0.962, and 0.965, respectively [20]. However, it still needs large instruments for data collection, limiting its application in field detection. By contrast, the visual sensor array is becoming increasingly popular. Visual sensor array technology is a kind of advanced sensing technology [21]. The advantages of simple operation, fast response, low cost, and convenient signal reading make it increasingly attractive to researchers [22]. Wu et al. designed a new method based on Fe_3_C/Fe-N-C catalyzing the oxidation of 3,3′, 5,5′ -tetramethylbenzidine (TMB) to produce blue oxidized TMB (oxTMB) reactive sensor for the detection of tea polyphenols and their monomers, but it solely relies on visible light signal changes in oxidized TMB (oxTMB), leading to limited discriminability for structurally similar catechin enantiomers. EGCG, EGC, ECG, and EC could be distinguished by the pattern recognition method at 50 μM concentration [23]. However, in food authenticity analysis, especially for products with subtle quality differences (e.g., adjacent-origin teas), most state-of-the-art dual-mode systems either focus on independent signal output (rather than synergistic signal enhancement) [24] or lack targeted design for key bioactive components (e.g., catechins in tea), resulting in insufficient sensitivity and specificity for practical detection [25]. As emerging optical materials [26], quantum dots (QDs) have the advantages of high fluorescence quantum yield, high photobleaching resistance, long fluorescence life, broad band absorption, narrow band emission, and good light stability [27]. QDs have promising applications in medicine, gene detection, food safety detection, and other fields [28]. On the other hand, porphyrin molecules, as large π-conjugated organic small molecules, have the characteristics of a wide spectral response range, fast response speed, and significant nonlinear absorption coefficient, and their derivatives are easy to modify, so they are a good optical material for the design and preparation of efficient sensors [29,30,31]. In addition, due to the introduction of the nano effect synergy, nano-modified porphyrins were endowed with better detection selectivity and sensitivity, as well as optical response and color rendering properties [32,33]. The composite sensing system constructed by QDs–nano porphyrins possesses the respective advantages of both materials, exhibiting excellent stability, substrate specificity, and selectivity, and showing great advantages in the fields of biocatalysis, biosensing, and food analysis [34,35]. Our group has established a dual-channel sensor array based on the “off” and “off-on” modes of the ZnCdSe QDs-KMnO_4_ system for the identification of 30 green teas originating from various species, grades, and origins, but with no detailed study on the quantification of mixed catechin enantiomers, the precise identification of Xihu Longjing from adjacent origins, the influence of storage time, and the quantitative analysis of quality factors [31,36,37]. Our group also has constructed a sensor for the specific recognition of chiral amino acids with simple structures in food using CdTe QDs-chiral nanoporphyrin, but quantitative analysis of mixed enantiomers was not conducted, making it unable to effectively analyze the authenticity of foods with slight differences, such as Xihu Longjing from adjacent origins [37,38]. Similarly, Liao et al. used dual-mode probes to quantitatively analyze the enantiomers of glutamine (Gln) and valine (Val) but did not conduct analysis of the mixed enantiomers and food authenticity either [39].

**Scheme 1:**
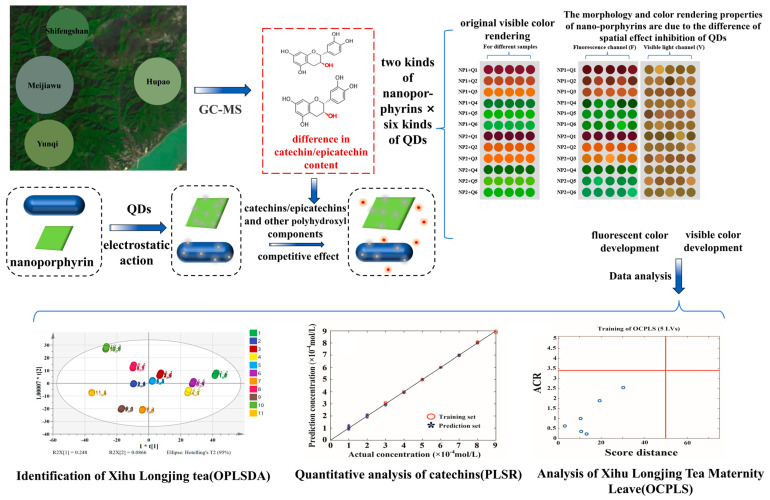
Schematic diagram of quantum dot–nano porphyrin dual-signal visual sensor.

The design strategy of the sensor array is shown in Scheme 1. In this study, a catechin-targeted nano-enhanced visual and fluorescent dual-mode sensor array was constructed using nano porphyrins and QDs for the analysis of Xihu Longjing from adjacent origins. Due to nano porphyrins that can enrich QDs on their surface and form a composite state, the catechin in Longjing teas will cause different degrees of inhibition in the process, resulting in various visual and fluorescent colors. Firstly, a quantitative analysis of the most representative catechin enantiomers (catechin, epicatechin, catechin gallate, and epicatechin gallate) [23]. Longjing tea is produced through the selective combination and utilization of sensing units. At the same time, each composition of two catechin enantiomers can be quantified, even if their mixing ratio is as low as 1:0.01, in real Longjing tea samples. Most importantly, through the accurate detection of such nuances, Xihu Longjing from adjacent origins, such as Shifengshan, Yunqi, Hupao, and Meijiawu, with various storage times was identified and was able to separate accurately, which further facilitated the successful identification of microtrace adulteration in Xihu Longjing. Finally, the flavor quality and antioxidant properties of Xihu Longjing could also be quantitatively evaluated by this sensor array. Due to the sensor array having good visual color rendering performance, the detection process is simple and stable; it provides new guidance for the design of sensor arrays for identifying minor differences in food authenticity and has certain application prospects not only in the analysis of Longjing teas but also in the whole tea industry.

## 2. Materials and Methods

### 2.1. Materials and Reagents

The diammonium 2,2-azino-bis 3-ethylbenzothiazoline-6-sulfonic acid (ABTS*) test kit was purchased from Biyuntian Biotechnology Co., Ltd. Sodium tellurite (97.0%), sodium hydroxide (NaOH, ≥98.0%), and tributylhexadecylphosphonium bromide (TPB, 98.0%) were purchased from Shanghai Aladdin Biochemical Technology Co., Ltd., Shanghai, China, 5,20-tetrad (4-nitrophenyl) porphyrin (NTPP) and 5,10,15,20-tetrad (4-manganese phenyl) porphyrin (MTPP) were synthesized in our laboratory. N-Acetyl-L-Cysteine (NAC, 99%), L-Glutathinone reduced (98%), mercaptosuccinic acid (98%), N, N-dimethylformamide (DMF), catechin, epicatechin, catechin gallate, epicatechin gallate, MgSO_4_, Na_2_SO_4_, K_2_SO_4_, CaSO_4_, FeSO_4_, HAS, and theanine were all analytically pure and acquired from Sahn Chemical Technology Co., Ltd., Shanghai, China. All experiments were performed with deionized water.

All Longjing tea samples were provided by the Hangzhou Tea Research Institute, China COOP, and the detailed information is listed in Table 1.

### 2.2. Preparation of CdTe QDs Modified with Different Ligands

Cadmium chloride (0.1650 g, 0.90 mmol) and mercaptoligand (1.2 mmol n-acetylcysteine, mercaptosuccinic acid ligand, or a mixture of the two in different ratios 1:1/3:1, reduced glutathione) were dissolved in 40 mL ultra-pure water, stirring at room temperature and pressure for 15 min. Adjust the pH to 9.70, stir with nitrogen at room temperature for 20 min, use a syringe to add sodium tellurite (0.0399 g, dissolved in 1 mL ultra-pure water), and finally add sodium borohydride (0.0540 g, dissolved in 1 mL ultra-pure water), continue to fill with nitrogen and stir for 15 min, and then quickly put into the reaction kettle. After hydrothermal reaction at 200 °C for 30 min (NAC ligand reaction for 40 min and 50 min, respectively, to obtain two kinds of QDs), an orange-yellow solution was obtained. The CdTe QDs solution was filtered through a 0.22 μM microporous filter membrane. The obtained CdTe QDs were stored in a refrigerator at 4 °C. Detailed information is listed in Table S1. Although CdTe QDs have certain toxicity, we use them in small amounts, with low toxicity. Moreover, we do not add them to food but take samples for testing. After use, the reagents will be uniformly recycled and processed, so there will be no toxic effects.

### 2.3. Preparation of Nano Porphyrins

Configure 40 mL of tributylhexadecyl phosphorus bromide aqueous solution with a concentration of 5.0 mmol/L, and adjust the pH value to 8. Then, the water bath was heated to 40 °C, and 8 × 10^−4^ mol/L of 5,10,15, 20-tetris (4-bromo-phenyl) manganese porphyrin (5,10,15, 20-tetris (4-aminophenyl) porphyrin) was dissolved in 1 mL of DMF, then dropped into the above aqueous solution, slowly stirred for 30 min, and stored in the refrigerator at 4 °C. The nano porphyrins synthesized by NH_2_ porphyrins are set as NP1, and the nano porphyrins synthesized by Mn porphyrins are set as NP2.

### 2.4. QDs–Nano Porphyrin Dual Signal Visualization Sensor Construction

The sensor array is composed of 12 sensing units. Sensing units M1 to M6 are Q1–Q6 (100 μL, 6 × 10^−9^ mol/L) mixed with 5,10,15,20-tetri (4-bromophenyl) manganese nanoporphyrin (100 μL, 1.6 × 10^−5^ mol/L), respectively. Sensing units N1 to N6 are Q1–Q6 (100 μL, 6 × 10^−9^ mol/L) composed of 5,10,15,20-tetri (4-aminophenyl) nanoporphyrin (100 μL, 1.6 × 10^−5^ mol/L). A certain volume of water was added to control the total system, which had a total volume of 300 μL, after each reaction hole was filled with detection samples. The corresponding visible light visualization is set as V, and the fluorescence visualization is set as F, as shown in Table 2.

### 2.5. Sample Treatment

A total of 0.05 g of dry Longjing tea samples were added into 10 mL of deionized water at 60 °C for 30 min and then filtered for later use. All the adulterated samples were prepared simultaneously and obtained under the same brewing conditions.

Longjing tea with different storage times: Put 4 g of each of 11 kinds of Longjing tea into sealed bags and incubated them for 5 days, 10 days, 20 days, 30 days, and 60 days, respectively, under the condition of 25 °C and 40% humidity in the incubator (MJP-80S, SENXIN, Shanghai, China.)

### 2.6. Sample Detection and Data Analysis

The fluorescence spectra were obtained by using a fluorescence spectrophotometer (F-7000, Hitachi, Tianjin, China). The nano porphyrins (8 × 10^−6^ mol/L), QDs (3 × 10^−10^ mol/L), certain concentrations of enantiomers (catechin and epicatechin, catechin gallate and epicatechin gallate), or 100 μL Longjing tea samples were added into the cuvette and fixed to 1 mL with ultra-pure water. The excitation wavelength was set as 340 nm.

The ultraviolet spectra were analyzed by using an ultraviolet spectrophotometer (U-3900, Hitachi, Japan). The nano porphyrins (8 × 10^−4^ mol/L), QDs (3 × 10^−8^ mol/L), certain concentrations of enantiomers (catechin and epicatechin, catechin gallate and epicatechin gallate), or 100 μL Longjing tea samples were added into the cuvette and fixed to 1 mL with ultra-pure water. The visible light channel requires the synergistic effect of QDs and NPs. High concentration (6 × 10^−9^ mol/L) can shorten the color development time (achieving stable color within 5 min), meeting the demand for “rapid detection”. The QD fluorescence intensity of the fluorescence spectrophotometer for fluorescence detection at 3 × 10^−11^ mol/L is 500–1200 au, which not only meets the requirement of “linear correlation between ΔF/F_0_ and concentration” (linear range 1 × 10^−11^–1 × 10^−7^ mol/L, R^2^ = 0.99), it also avoids the “self-quenching” caused by high-concentration QDs.

For visual detection, QDs, nano porphyrins, and samples to be tested are added to each 96-well plate in order. To control the influence of adding time on the reaction, all samples were added using an Eppendorf multi-channel pipette. First, two pairs of enantiomeric catechins and epicatechin, catechin gallate and epicatechin gallate, were diluted in a series of concentrations including 1 × 10^−3^, 7 × 10^−4^, 5 × 10^−4^, 3 × 10^−4^, 1 × 10^−4^, 5 × 10^−5^, 1 × 10^−5^, 1 × 10^−6^, and 1 × 10^−7^ mol/L. Then, 200 μL of sensor solution and 100 μL of enantiomer solution with different concentrations were added successively to the corresponding 96-well plate locations. When the last sample was added, it was mixed evenly at room temperature and reacted for 15 min. Then, the 96-well plate was placed on a shadowless bottom lamp and an ultraviolet lamp and photographed with an iPhone 16 Pro in the darkroom. The camera parameters of the mobile phone were adjusted to a shutter speed of 1/50 s (the mobile phone was fixed in the top slot of the fixture, and the center of the lens was aligned with the center of the array). ISO was 100 (the lowest native ISO, reducing noise); the white balance was customized (calibrated with a standard gray card). This process recorded the fingerprint of the characteristic value of each sensing unit, and parallel experiments were repeated six times. To understand the different responses of the array sensor, after taking the picture, the R, G, and B values were extracted using Photoshop (CS6, SAN Jose, CA, USA). These differences were then amplified by a factor of 10 to create a color difference map. Partial least squares regression (PLSR) model was performed in MATLAB software (Version 2019b) based on the original RGB values of the screened sensing units in the color difference plots [40]. Only the data of the color difference map has been amplified for better visualization effect; all data processing directly uses the original data.

To demonstrate the high selectivity of the sensor array, two catechin enantiomers, catechin and epicatechin, and catechin gallate and epicatechin gallate were mixed, respectively, in different proportions. For the concentration ratios of catechins to epicatechins set as 1:1, 1:0.1, and 1:0.01, 200 μL of sensing point solution, 100 μL of catechin solution (3 × 10^−4^ mol/L), and 100 μL of epicatechin solutions of different concentrations were successively added to the corresponding 96-well plate locations. For the concentration ratios of catechins to epicatechins set as 1:9, 2:8, 3:7, 4:6, 5:5, 6:4, 7:3, 8:2, and 9:1, 200 μL of sensing point solution and 100 μL of enantiomer solution (total concentration fixed as 3 × 10^−4^ mol/L) were added to the corresponding position of the 96-well plate. After mixing for 15 min, fingerprint images for each concentration were recorded six times in parallel under a shadowless bottom lamp and an ultraviolet lamp. Then, the color change in the sensor array response information was recorded. After filtering the obtained information, PLSR was used for quantitative analysis in MATLAB software (Version 2019b). Another set of catechin gallate and epicatechin gallate was dealt with as above.

To analyze the authenticity of tea, 11 kinds of Xihu Longjing from adjacent origins were used instead of the enantiomers. The color difference was mapped and screened, and the screened sensing units were dealt with by orthogonal partial least squares discriminant analysis (OPLS-DA) in Simca software (Version 14.1).

To identify the adulteration of Longjing tea, Shifengshan (super grade), Wuniuzao, and Meijiawu (second grade) teas were selected for the purpose of identifying tea adulteration. First of all, the two cases of tea adulteration were artificially configured. The volume ratio of real samples to fake samples was set as 1:1, 1:0.1, 1:0.01, and 1:0.001, and 200 μL of sensing point solution, 100 μL of real samples, and 100 μL of fake samples with different concentrations were successively added to the corresponding position of the 96-well plate. The volume ratio of real samples to fake samples was set as 9:1, 8:2, 7:3, 6:4, and 5:5, and 200 μL of sensing point solution and 100 μL of tea solution (including real samples and fake samples) were added to the corresponding positions of the 96-well plate, respectively. After mixing for 15 min, the fingerprint images for each concentration were recorded nine times in parallel under shadowless and ultraviolet lamps. The color change response information of the sensor array was then recorded. The results were drawn into color difference plots and filtered and then used for the one-class partial least squares method (OCPLS) performed in MATLAB software (Version 2019b).

**Scheme 2:**
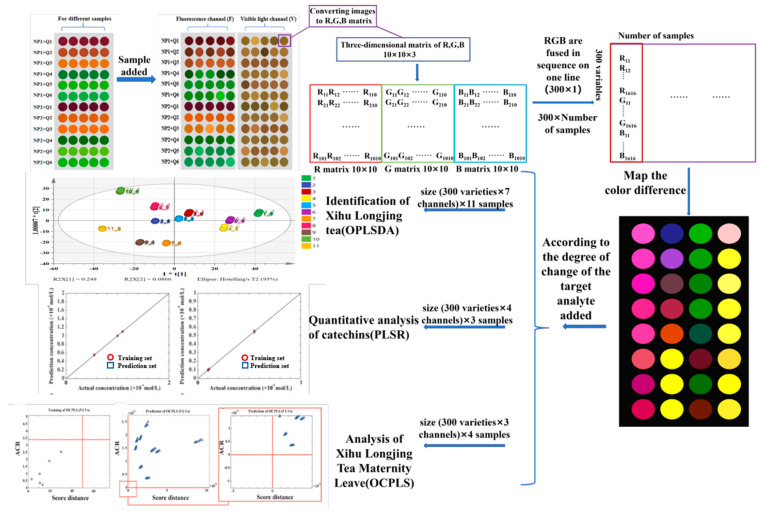
Schematic diagram of QDs–nano porphyrin dual-signal visual sensor.

The data analysis principle was illustrated in Scheme 2. The picture of each sample spot in the photos was collected separately and transformed into a picture of 10 × 10 pixels through the extract color blocks through the image cropping function of Photoshop. Then, the picture was processed with the Matlab imread command, and three data arrays corresponding to the RGB value were obtained (the size of each data array is 10 × 10, which contains 100 variables), and then the values of 300 variables in the three data arrays are rearranged and combined into a vector. Each sample is represented by a vector contained within (300 × channel number) by 1. These variables are further fused according to the class group of the sample; the color difference calculation is obtained by adding the sample matrix–blank sample matrix (diff_A = Sample(2:end) − Sample(1)), and finally calculated by PLSR, OCPLS, and OPLSDA models.

### 2.7. Antioxidant Test of Longjing Tea

The total antioxidant capacity (T-AOC) assay kit (ABTS* method) was used to determine the antioxidant capacity of tea. Tea contains antioxidants, and we tested them. ABTS oxidizes to green ABTS* under the action of appropriate oxidants, and the production of ABTS* is inhibited in the presence of antioxidants. According to the requirements of the kit, 734 nm/414 nm can be selected. We chose to test at 414 nm. The total antioxidant capacity of the sample can be determined by measuring the absorbance of ABTS* at 414 nm. ∆A was used to evaluate the antioxidant activity capacity, where Acontrol and Atest were the absorbance of ABTS* at 414 nm before and after the addition of tea samples:

∆A = Acontrol − Atest

### 2.8. Sensory Evaluation

In this study, the aroma (flavor type, purity, intensity, and persistence) and taste (sweetness, bitterness, and freshness) of 11 Longjing teas were evaluated, and the conclusions were drawn according to professional tea evaluation terms and scoring methods.

Sensory evaluation was performed by a tea sensory evaluation expert group from the National Tea Quality Inspection Center, China; the Hangzhou Tea Research Institute of the All-China Federation of Supply and Marketing Cooperatives; and et al. (including three males and three females, aged 30–40 years). The color, aroma, and taste of tea infusions were evaluated in accordance with the Methods for Sensory Evaluation of Tea (GB/T 23776–2018) [41]. The aroma and taste of the tea samples were evaluated using a 100-point scale (with 0–20 indicating zero intensity, 40–60 indicating medium intensity, and 80–100 indicating high intensity) for each sensory attribute.

### 2.9. Determination of Catechin Compounds in Tea by HPLC-MS/MS Method

Equipment: liquid chromatography-mass spectrometry (LC-MS) equipment: AB SCIEX 4500 LCMSMS (AB SCIEX, Boston, MA, USA), ODS chromatographic column (2.0 × 150 mm, actual detection uses shim-pack XR-ODS 150 mm*2 mm).

Pretreatment equipment: vortex mixer (IKA Company, Staufen, Germany), KQ-500DE CNC ultrasonic cleaner (Kunshan Ultrasonic Instrument Co., Ltd., Kunshan City, China), PWN224ZH/E electronic analysis platform (Ohaus Instruments (Changzhou) Co., Ltd., Changzhou, China), high-speed refrigerated centrifuge (Shanghai Luxiangyi Centrifuge Instrument Co., Ltd., Shanghai, China).

Mobile phase and matrix: 0.3% formic acid aqueous solution (1000 mL Watsons water + 3 mL formic acid, mix well and ultrasonicate for 2 min, valid for 1 week); RO1 (90 mL Watsons water + 210 mL methanol, mix well and ultrasonicate for 2 min, valid for 1 month); RO2 (20 mL Watsons water + 20 mL methanol, mix well and ultrasonicate for 2 min, valid for 1 month).

Liquid phase parameter settings: column temperature 45 °C, mobile phase A is 0.3% formic acid aqueous solution, mobile phase B is methanol, total flow rate 0.3000 mL/min, injection volume 2 μL; operate in accordance with the prescribed time program (0.10 min: A75%, B25%; 10.0 min: A50%, B50%, etc.) and the switching valve program (0.1 min for B and 12.0 min for A).

Mass spectrometry parameter settings: ionization mode ESI-, detection mode MRM; Curtain Gas 35.0, Collision Gas 9.00, IonSpray Voltage-4500, etc., (for details, see the parameters in the table); the four target substances were detected according to parameters such as the precursor m/z and product m/z (catechin: 289.1→109.0, etc.), and the methods and standard curves were stored in the catechin folder.

All the HPLC-MS/MS results were provided by Spectro (Wuhan) Medical Biotechnology Co., Ltd., Wuhan, China.

## 3. Results

### 3.1. Sensor Units Characterization

**Figure 1:**
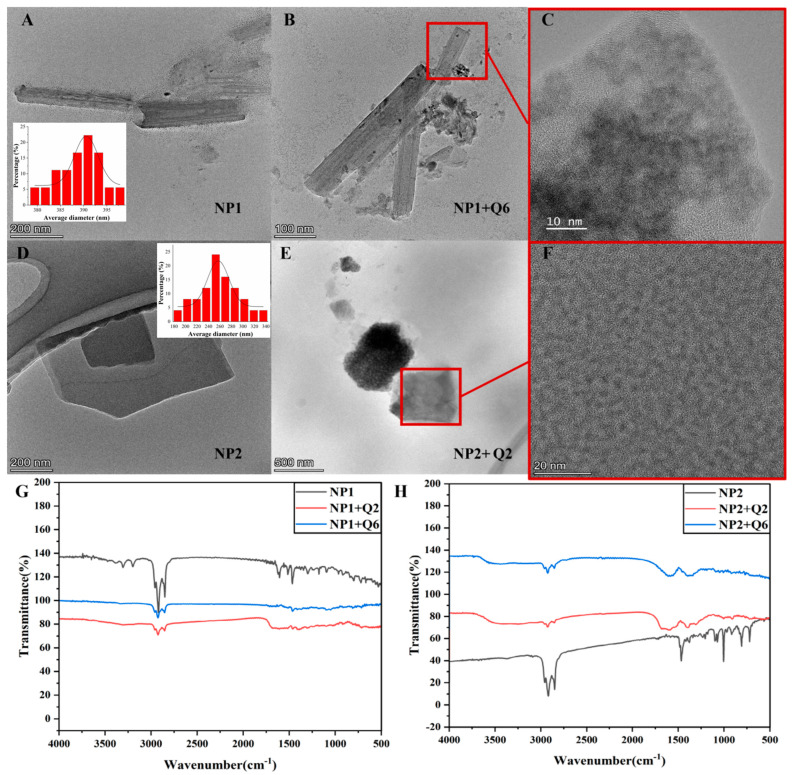
Morphology and structure characterization of nanoporphyrins by TEM and IR. TEM (**A**–**C**) morphological changes in NP1 after adding Q6. TEM (**D**–**F**) morphological changes in NP2 after adding Q2. IR (**G**) structural changes in NP1 after adding Q2 and Q6. IR (**H**) structural changes in NP2 after adding Q2 and Q6.

Since the surface of the QDs is negatively charged, and the surface of the nanoporphyrins is covered by the positively charged surfactant, nanoporphyrins can enrich QDs on its surface and form a composite state. Therefore, the optical properties of the nanoporphyrins will change after the addition of QDs [42]. In order to investigate this change, nanoporphyrin–QDs complexes were selected for transmission electron microscope (TEM) and infrared spectral characterization (IR). TEM images of six kinds of QDs, nanoporphyrins, and complexes are shown in Figure 1 and S1. The quantum dots of the sensor have a spherical shape, and the particle sizes of Q1-Q6 are approximately 2 nm, 2 nm, 2 nm, 1.8 nm, 1.8 nm, and 2.8 nm, respectively. NPs self-aggregate and exist in the form of aggregates. The length of each monomer of NP1 is approximately 380 nm–400 nm, and that of each monomer of NP2 is approximately 180 nm–340 nm. As for NP1, its original appearance is rod-like nanomaterial, and the original appearance of NP2 is lamellar. After the addition of QDs, it can be clearly seen that the QDs accumulate on the surface of the nanoporphyrin, verifying the rationality of the sensor array design. As shown in Figure 1G,H, after adding different QDs, the infrared spectrum of nanoporphyrins also changed significantly. Specifically, it can be seen from Figure 1G that after adding different QDs, the vibration amplitude at the saturated C-H bond energy at about 2900 cm^−1^ decreases, the NP1 + Q6 peak is shallower, and the transmittance is higher, while at about 1500 cm^−1^, the vibration wave number at C-N/C-O also decreases due to molecular forces, and NP1 + Q2 is shallower. It can be found in Figure 1H that after adding different QDs, a wide and slow O-H peak due to hydrogen bonding occurs at about 3500 cm^−1^, the vibration amplitude at the saturated C-H bond energy decreases at about 2900 cm^−1^, NP2 has obvious absorption in the C=O peak range at about 1550 cm^−1^, and weakens after adding QDs. However, at around 1050 cm^−1^, the number of vibration waves at C-N/C-O also decreases. It can be seen from Figure S2 that the potential of QDs changes from negative to positive after the addition of NPs. The different interactions between QDs and nano porphyrins were further verified. Rhodamin 6G was used as a standard for determining PL quantum yields (QYs), and the QYs for CdTe QDs was 33 (Figure S3). The fluorescence intensity only decayed by 12.6% after a period of up to 4000 s, indicating that the quantum dots used have excellent fluorescence properties.

**Figure 2:**
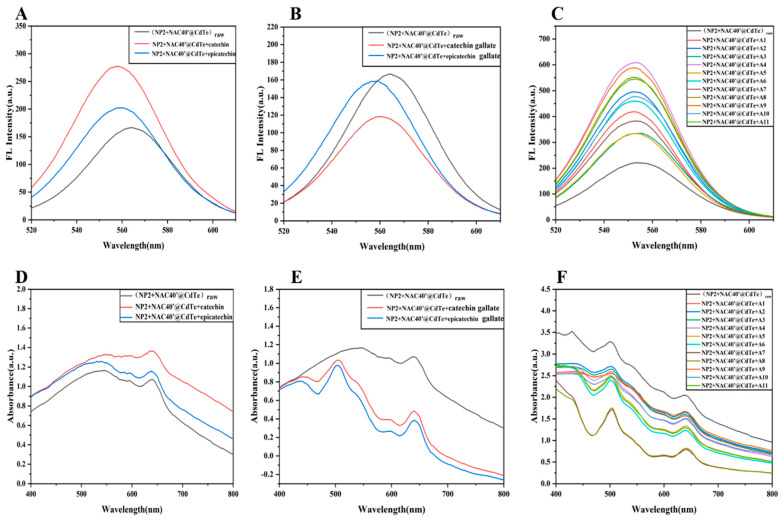
Fluorescence and UV spectral characterization of NP2 + Q3 after the addition of enantiomers or Longjing tea. (**A**) Fluorescence spectral response of NP2 + Q3 to catechins and epicatechin (1 × 10^−5^ mol/L). (**B**) Fluorescence spectral response of NP2 + Q3 to catechin gallate and epicatechin gallate (1 × 10^−5^ mol/L). (**C**) Fluorescence spectral responses of NP2 + Q3 to 11 different types of Xihu Longjing teas. (**D**) Ultraviolet spectral responses of NP2 + Q3 to catechins and epicatechins (1 × 10^−5^ mol/L). (**E**) Ultraviolet spectral responses of NP2 + Q3 to catechin gallate and epicatechin gallate (1 × 10^−5^ mol/L). (**F**) Ultraviolet spectral responses of NP2 + Q3 to 11 different types of Xihu Longjing teas.

In order to further test the recognition performance of the sensor array on the structurally similar components such as enantiomers and the authenticity of Longjing tea, 1 × 10^−5^ mol/L of two catechin enantiomers, including catechin and epicatechin, catechin gallate and epicatechin gallate, and different Longjing tea samples were selected and added into the units of NP1 + Q5 and NP2 + Q3. As shown in Figure 2 and Figure S4, after the addition of 1 × 10^−5^ mol/L catechin and epicatechin, the fluorescence and UV spectra of the sensor units were changed, and there were obvious differences between the enantiomers. This may be due to the difference in steric hindrance between enantiomers, which leads to the difference in binding ability with the chiral ligands on the QD surface (e.g., NAC, GSH) and nano porphyrins of QDs [38]. What is more, even for Xihu Longjing from adjacent origins with small composition differences, there are significant differences in the optical response of sensing units, which indicates that the sensor units can accurately detect enantiomers and accurately identify Xihu Longjing from adjacent origins through fluorescence and visible signal response. The ultraviolet absorption peaks of catechin compounds are between 250 and 350 nm, while the fluorescence emission peaks of QDs–NPs are located between 520 and 680 nm. The ultraviolet absorption peak (nm) of catechin compounds does not overlap with the fluorescence emission peak (nm) of QDs–NPs, so FRET and IFE effects cannot occur [43].

### 3.2. Precise Detection of Two Catechin Enantiomers

To verify the sensor unit’s detection performance for trace and structurally similar components, we selected the two most representative catechin enantiomers in Longjing tea—catechin and epicatechin, catechin gallate and epicatechin gallate (Figure S5)—for quantitative analysis. The catechin enantiomers were further quantitatively analyzed by fluorescence detection. The (5 × 10^−6^–1 × 10^−8^ mol/L) catechins were analyzed by fluorescence spectra with the Q3 + NP2 channel and linearly analyzed by the Stern–Volmer equation, as shown in Table S2 and Figure S6, where C is the concentration of the analyte and K_SV_ is the quenching constant. F_0_ represents the original fluorescence intensity of the fluorescence channel, and F represents the fluorescence intensity when enantiomers are added. Due to the fact that the larger the K_SV_, the stronger the binding ability, which can be accurately recognized by the sensor channels. From the results, it is known that the detection limits of catechin, epicatechin, catechin gallate, and epicatechin gallate were calculated by 3 N/S (N was the relative standard deviation, while S was the slope) as 2.5 × 10^−9^, 2.2 × 10^−9^, 7.5 × 10^−9^, and 8.9 × 10^−9^ mol/L, respectively. However, the linearity is not very good, and the quantification is inaccurate; a more accurate analytical method needs to be established:

F_0_/F = 1 + K_SV_/C

**Figure 3:**
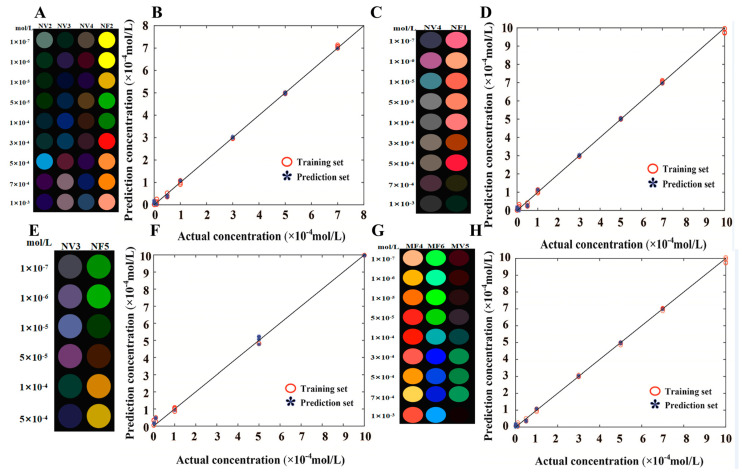
The color difference maps and quantitative analysis results of visual sensor arrays with different concentrations of catechin and epicatechin, catechin gallate and epicatechin gallate: the different concentrations of catechin from 1 × 10^−3^ mol/L to 1 × 10^−7^ mol/L (**A**) and quantitative analysis result (**B**). The different concentrations of epicatechin from 1 × 10^−3^ mol/L to 1 × 10^−7^ mol/L (**C**) and quantitative analysis result (**D**). The different concentrations of catechin gallate from 5 × 10^−4^ mol/L to 1 × 10^−7^ mol/L (**E**) and quantitative analysis result (**F**). The different concentrations of epicatechin gallate from 1 × 10^−3^ mol/L to 1 × 10^−7^ mol/L (**G**) and quantitative analysis result (**H**).

The results are presented in Figure 3. The visible light channels NV2, NV3, and NV4 and fluorescence channel NF2 were selected according to color change response and recombined for catechin quantitative analysis. After the introduction of the PLSR model, precise quantification of four catechins was successfully achieved. The core results are summarized in Table 3. The optimal latent variables were verified by seven-fold interaction for catechin, epicatechin, catechin gallate, and epicatechin gallate as six, seven, five, and six, respectively. Herein, the random seed was set as 12, and 54 samples were randomly divided into 30 training samples and 24 prediction samples. The corresponding RMSEC was calculated as 0.003, 0.055, 1.24 × 10^−4^, and 0.0028, respectively. The RMSEP was 0.004, 0.06, 1.28 × 10^−4^, and 0.004, respectively, and the RNSE was 0.104, 0.099, 0.509, and 0.003, respectively. The linear fitting equations are, respectively, y = 1.007x + 0.02, y = 1.009x − 0.004, y = 1.001x + 0.014, and y = 1.000x − 0.0003.

It can be seen from Table 3 that the detection range of the four catechins covers 1 × 10^−7^ to 1 × 10^−3^ mol/L, and all maintain a high coefficient of determination of 0.99, indicating that the quantitative linear relationship of the sensor array is excellent. The recovery rate ranged from 94.8% to 104.3%, verifying the accuracy of the method.

Due to the different proportions of enantiomers in different Longjing teas, it is necessary to analyze two catechin enantiomers, including catechin and epicatechin, and catechin gallate and epicatechin gallate mixed in different proportions. Quantitative analysis results of each component in the enantiomer mixture are shown in Figure S8. The concentration ratios of catechin to epicatechin and catechin gallate to epicatechin gallate were set at 1:1, 1:0.1, and 1:0.01, respectively, first. The visible light channel NV1 and the fluorescent channel NF1, as well as the visible light channel MV6 and the fluorescent channel MF1, were recombined for quantitative analysis. On the other hand, the concentration ratio of catechin enantiomers was set as 1:9 to 9:1, and quantitative analysis was conducted on the recombinant visible light channels NV1, NV6, and fluorescence channel NF1 (Figure S7). The optimal latent variable verified by seven-fold interaction verification for the catechin mixture ratio of 1:1–1:0.01 was three. The random seed was set to 12, and 18 samples were randomly divided into 12 training samples and six prediction samples. The RMSEC for the catechin mixture was 9.29 × 10^−4^ and 9.06 × 10^−4^, respectively. The RMSEP was 7.67 × 10^−4^ and 7.37 × 10^−4^, respectively, and the RMSE was 0.0065 and 0.0631, respectively. The linear fitting equations are, respectively, y = 1.095x − 0.057 and y = 1.3227x + 0.024. On the other hand, the optimal latent variable obtained by seven-fold interaction verification for the mixed ratios of catechins 1:9 to 9:1 was eight. The random seed was set to 12, and 36 samples were randomly divided into 24 training samples and 12 prediction samples. The RMSEC was 4.77 × 10^−4^ and 4.78 × 10^−4^, and the RMSEP was 5.7 × 10^−4^ and 5.75 × 10^−4^, respectively. The RMSE was 0.0681 and 0.0774, respectively, and the linear fitting equations were y = 0.989x + 0.006 and y = 1.006x + 0.0004. For the catechin gallate and epicatechin gallate mixture, the recombination of the visible light channels NV4 and NV5 and the fluorescence channel NF1 was quantitatively analyzed (Figure S9), and the optimal latent variable was verified as three by seven-fold interaction verification for a catechin gallate mixture ratio of 1:1–1:0.01. The RMSEC was 9.29 × 10^−5^ and 9.06 × 10^−5^, respectively; the RMSEP was 7.67 × 10^−5^ and 7.37 × 10^−5^, respectively; and the RMSE was 0.0065 and 0.0631, respectively. The linear fitting equations were y = 1.095x − 0.057 and y = 1.3227x + 0.024. The optimal latent variable for mixed ratios of catechin gallate 1:9 to 9:1 was calculated as eight, RMSEC was 4.77 × 10^−15^ and 4.78 × 10^−15^, and RMSEP was 5.7 × 10^−15^ and 5.75 × 10^−15^, respectively. The RMSE was 0.0681 and 0.0774, respectively, and the linear fitting equations were y = 0.989x + 0.006 and y = 1.006x + 0.0004. In addition, this sensor array was also applied to the quantitative analysis of enantiomers in 11 types of Longjing tea. Compared with the concentration obtained by HPLC-MS, the recovery rate can reach 94–106%, indicating that the quantum dot–nanoporphyrin sensor array has excellent detection performance, laying a foundation for the identification and precise analysis of Xihu Longjing tea from adjacent production areas.

### 3.3. Precise Discrimination of 11 Kinds of Xihu Longjing from Adjacent Origins

**Figure 4:**
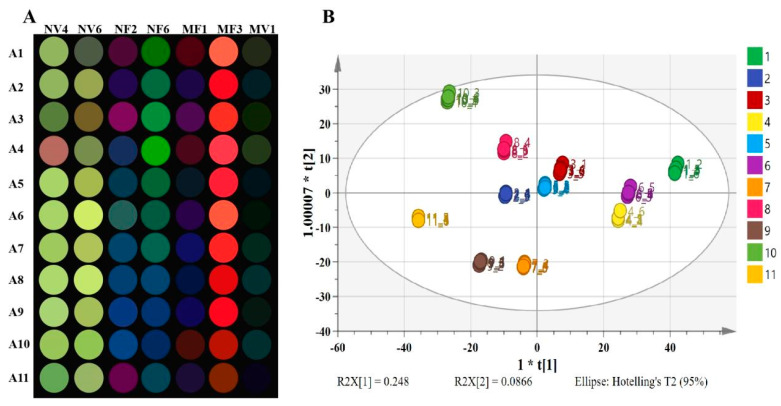
Discrimination results of 11 kinds of Xihu Longjing from adjacent origins: (**A**) The color difference map of visual sensor array; (**B**) linear discrimination results via OPLSDA.

Eleven different grades of Xihu Longjing from adjacent origins were selected to investigate the detection performance of the array. The color change results as shown in Figure 4A and Figure S10, the visible light channels NV4, NV6, and MV1 and the fluorescence channels NF2, NF6, MF1, and MF3 were recombined and used for quantitative analysis and the linear discrimination results of OPLSDA as shown in Figure 4B. Through the OPLSDA results [44,45], different Xihu Longjing can be accurately separated with R^2^X = 0.99, R^2^Y = 0.998, Q^2^ = 0.004, RMSEE = 0.007, and RMSECV = 0.01. It can be observed that the sensor array can accurately distinguish Xihu Longjing from adjacent origins. The application range of the nano-porphyrin composite system in Longjing tea identification was expanded, which laid a foundation for the subsequent application. As shown in Figures S11 and S12 and Table S4, the OPLS-DA results indicate that there are significant differences among the Xihu Longjing tea samples. The 999 permutation tests indicate that there is no overfitting and demonstrate the accuracy of the classification results.

The quality of Longjing teas can change with the corresponding storage time. In order to monitor this process, 11 different Longjing teas were cultured in incubators for 5, 10, 20, 30, and 60 days, respectively, and the change in Longjing tea quality was monitored using the sensor array. The color change results are shown in Figures S13–S16; the visible light channels NV4, NV6, and MV1 and the fluorescence channels NF2, NF6, MF1, and MF3 were recombined for precise analysis. From the color response information, the quality of tea changed with the storage time, and the degree of change varied with the quality of Longjing tea. With the increase in tea quality, the quality change is less obvious to storage time, showing as the minor color changes in the sensor array. For example, Meijiawu second grade began to have obvious color change between 5 and 10 days, while Shifengshan tea was between 30 and 60 days. In addition, after the introduction of the PLSR model, PLSR quantitative analysis results are shown in Table 4. It can be seen from the PLSR results that in all cases, different storage times of Longjing teas can be quantitatively monitored. Different heating temperatures and antioxidants will also cause varying degrees of impact. As shown in Figure S19, tea steeped at 60 °C for 30 min was used as the control group, and tea steeped at 80 °C for 3 min was compared with it. From the color difference in the 96-well plate, it can be seen that the effect of tea steeped at 60 °C is more obvious. This is because 80 °C can rapidly destroy the cell walls of tea leaves and promote the dissolution of enantiomers such as catechins. Based on the optimal extraction conditions (60°/30 min), antioxidant AA was added. The control group without AA was found to have a better effect from the 96-well plate. This is due to the intensity of AA’s non-specific interference (ligand destruction, competitive binding). It exceeded its protective effect on catechin oxidation—although AA could reduce catechin oxidation by 15%, the sensor signal distortion and enantiomer recognition deviation introduced thereby were more severe, ultimately resulting in the experimental group’s effect being inferior to that of the control group. The sensor characteristics were associated with HPLC-MS catechin enantiomers and indeed changed (Table S6).

### 3.4. Authenticity Identification of Longjing Tea

**Figure 5:**
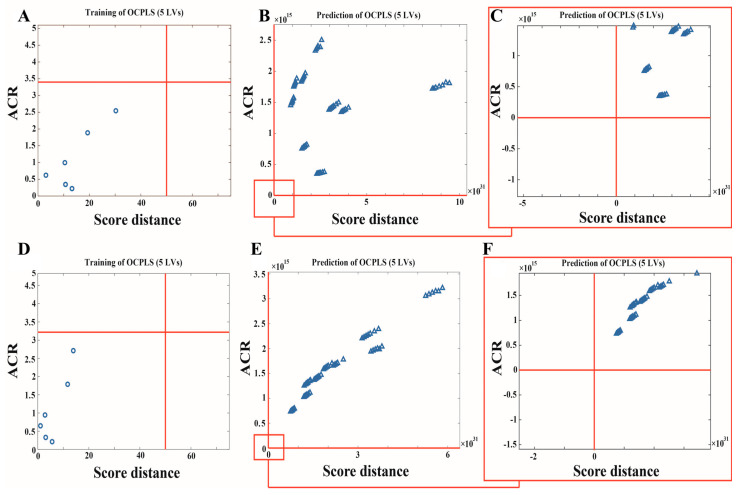
Adulteration assessment of Longjing teas from Shifengshan mixed with Wuniuzao via OCPLS: (**A**) the result for training samples; (**B**) the result for prediction samples; (**C**) the enlarged view of the regular point region in the result. Adulteration assessment of Longjing teas from Shifengshan mixed with Longjing teas from Meijiawu via OCPLS: (**D**) the result for training samples; (**E**) the result for prediction samples; (**F**) the enlarged view of the regular point region in the result.

A catechin-targeted, nano-enhanced visual and fluorescent dual-mode sensor array as constructed can also realize the distinction of adulterated Longjing tea. Due to Wuniuzao and Longjing tea being very similar in appearance and processing technology, Wuniuzao is often used as an adulterant in high quality Longjing tea. Herein, Longjing tea from Shifengshan in super grade blended with different ratios of Wuniuzao was set as an example to evaluate the feasibility of this visual array sensor. The visible light channels NV4 and NV5 and the fluorescence channel NF5 were recombined and used for authenticity identification of Longjing tea from Shifengshan in super grade mixed with Wuniuzao with the ratio ranging from 1:1 to 1:0.001 and 9:1–5:5 (Figure 5A,B). Unlike traditional quantitative models or classification models, OCPLS only focuses on the matching degree between unknown samples and corrected samples. Therefore, it can accurately identify adulterated samples of different degrees, but it cannot analyze the adulteration amount of adulterated samples. After the introduction of the OCPLS model, every adulterated sample was correctly identified. The validation ratio of the optimal latent variable through leave-one-out cross is five (Figure S20). Six real samples were taken as the training set, with six samples each of 1:1, 1:0.1, and 1:0.01, totaling 48 samples, as the prediction set. On the other hand, for adulteration of different qualities that are more difficult to identify, Longjing tea from Shifengshan in super grade blended with different ratios of Longjing tea from Meijiawu in second grade was selected as an example. The visible light channels NF1 and NF2 and the fluorescence channel NV3 were recombined and used for authenticity identification of Longjing tea from Shifengshan in super grade mixed with Meijiawu in second grade with the ratio ranging from 1:1 to 1:0.001 and 9:1–5:5 (Figure S20C,D). Similarly, all adulterated samples, even those with an adulteration ratio as low as 0.001, can be accurately identified. At the same time, black tea was made using adulterated channels, and the results are shown in Figure S17, verifying that it can also be used to distinguish other types of tea. The results of the 1:001 adulteration samples from other batches were also accurately predicted by OCPLS (Figure S18).

### 3.5. Quantitative Evaluation of the Antioxidant Activity and Flavor of Longjing Teas

**Figure 6:**
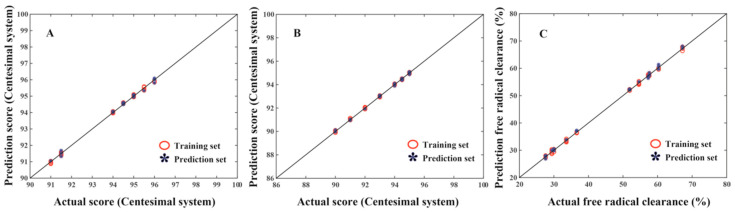
The result of the antioxidant activity and flavor of Longjing teas via PLSR: (**A**) quantitative analysis of antioxidant activity of Longjing teas; (**B**) results of quantitative analysis of aroma of Longjing teas; (**C**) results of quantitative analysis of relish of Longjing teas.

For phenolic hydroxyl groups with rich flavors, the entire molecule has electron-rich characteristics; thus, they are prone to bond with positively charged materials and interact with them. According to the potential diagram (Figure S2), the surface of QDs is negatively charged, while that of NPs is positively charged. When polyphenolic components rich in phenolic hydroxyl groups are added, they will combine with positively charged NPs, thereby causing fluorescence changes in the QDs–NPs complex system. As for amino acid-based flavor components, we have reported that the QDs–NPs system can interact with them and produce fluorescence changes [46]. Based on the above phenomena, it indicates that the QDs–NPs composite sensing array we established can generate differential responses to flavor components such as polyphenols and amino acids in Longjing tea, and then conduct quantitative analysis of its flavor quality [47]. As key factors, flavor quality, taste quality, and antioxidant activity are often used to evaluate the quality characteristics of Longjing tea. The antioxidant results of Longjing teas are shown in Table 1. The visible light channels NF2, NF6, MF1, and MF3 and the fluorescence channels NV4, NV6, and MV1 were selected and combined with the PLSR model; the antioxidant activity of each Longjing tea could be accurately quantified with a linear correlation coefficient of 0.99 (Figure 6). As the random seed was set as 12, 66 samples were randomly divided into 44 training samples and 22 prediction samples. The latent variables of aroma quality and relish quality were five and four, respectively, by seven-fold interaction verification, and RMSEC were 0.1043 and 0.1616, respectively. The RMSEP was 0.1373 and 0.2036, respectively; the linear fitting equations were y = 1.002x − 0.0316 and y = 0.9932x − 0.0019, respectively, and the RMSE was 0.1402 and 0.2018, respectively. On the other hand, the latent variable of antioxidant quality was calculated as 7, RMSEC was 0.0017, and RMSEP was 0.0017. The linear fitting equation is y = 1.003x − 0.0095, and RMSE is 0.1402. In addition, the results of sensory evaluation are shown in Table S5. The same sensing channel was used in the quantitative evaluation of Longjing tea flavor and taste, and the flavor quality score and taste quality score were accurately quantified by the PLSR model (Figure 6). Compared with other sensors, this sensor can achieve the distinction of tea origin, the authenticity of adulteration, and the quantitative detection of catechin enantiomers (Table 5) [25,48,49]. Among them, × indicates that it was not done, and ✓ indicates that it was done.

### 3.6. Stability and Anti-Interference Ability of Composite Colorimetric Sensor

To verify that the sensor array constructed in the study has favorable stability, the solution with the pH value ranging from four to 11 was added into each sensing unit, leaving the remaining methodological steps unchanged. After mixing for 15 min, the fingerprint of each sensor unit was recorded. The color difference diagram is shown in Figure S21A,B. As shown in the figure, the QDs–NPs composite sensor array can maintain good stability.

Meanwhile, to ascertain the anti-interference performance of the composite sensor array, high concentrations (1 mg L^−1^) of common metal ions and amino acids (Mg^2+^, K^+^, Ca^2+^, Fe^2+^, Gly, Glu, Ala, L-Theanine, HSA, HCO_3_^−^, CO_3_^2−^) were added to the detection system as interference components. The experimental procedure is the same as above. After mixing for 15 min, the fingerprint of each sensing unit is recorded, and the color difference diagram is shown in Figure S21C,D. The composite sensor can still produce a stable color response. The results show that the sensor array constructed in this study has good stability and anti-interference, which lays a foundation for the practical application of tea quality detection. The K_A, B_ values of catechin, epicatechin, and epicatechin gallate can be obtained from the anti-interference color difference graph data, which were 0.0016–0.07, 0.0016–0.015, 0.0011–0.013, and 0.001–0.011, respectively.

To prove that the QDs–NPs sensor is stable, five records were made in intra-day and inter-day. Photos were taken to extract the RGB values and draw a color difference graph, as shown in Figure S22. The results show that the QDs–NPs sensor is stable and repeatable. The intra-day and inter-day errors were 0.3–2.93% and 1.08–7.6%, according to relative standard deviation, respectively.

## 4. Conclusions

In this study, a catechin-targeted nano-enhanced visual and fluorescent dual-mode visual sensor array was constructed by nano porphyrins (NPs) and QDs for quantitative analysis of catechin enantiomers (catechin, epicatechin, catechin gallate, and epicatechin gallate) with the limit of quantification of 1 × 10^−7^ mol/L in Longjing tea through selective combination and utilization of sensing units. Simultaneously, the mixture of either catechin and epicatechin or catechin gallate and epicatechin gallate can be accurately analyzed even at a low ratio of 1:0.01. The catechin content in real Longjing tea samples could also be quantified with a recovery rate of 101.19 ± 0.03%. Otherwise, the subdivision of 11 kinds of teas and the visual detection of tea adulteration ratios as low as 1:001 identification can also be accurately separated, which thoroughly verified the sensor array’s excellent property. Most importantly, the flavor quality and antioxidant properties of Longjing teas could also be quantitatively evaluated by this sensor array. The rapid and effective dual signal visualization sensor selectively selects different channels according to the demand and provides an effective research strategy for rapid quality analysis of tea. At present, the long-term stability of the sensor needs to be improved: in an environment with a humidity greater than 60%, the fluorescence intensity of quantum dots will decrease. This detection range has certain limitations, only targeting the four main catechin enantiomers in Xihu Longjing tea. For oxidation products such as theaflavins and theabrownins in other teas (such as black tea), the targeted recognition unit needs to be redesigned. The design logic and technical framework of this sensor can be easily transferred to other high-value tea detection scenarios with “origin dependence and significant quality stratification”. In addition to tea, the “targeted recognition + multi-signal coordination” design concept of this dual mode sensor array can be extended to other high-value food fields that are prone to adulteration and quality stratification.

## Figures and Tables

**Table 1:** Longjing tea information used in this experiment.

Number	Label	Samples	Grades	Aroma	Taste Score	Total Antioxidant Capacity (∆A, ABTS mmol TE/L)
1	A1	Sh FRET ng	Super	94.5	95.0	0.1
2	A2	Shifeng	First	91.5	90.0	0.122
3	A3	Meijiawu	Super	95.0	95.0	0.192
4	A4	Meijiawu	First	94.0	93.0	0.201
5	A5	Meijiawu	Second	91.5	91.0	0.224
6	A6	Hupao	Super	96.0	94.5	0.173
7	A7	Hupao	First	95.5	94.0	0.182
8	A8	Hupao	Second	95.0	93.0	0.191
9	A9	Yunqi	Super	94.5	94.5	0.092
10	A10	Yunqi	First	94.0	93.0	0.098
11	A11	Yunqi	Second	91.0	92.0	0.112
12	A12	Wuniuzao	/	91.0	90.0	/

**Table 2:** Composition and detection conditions of each channel in the sensor array.

Channel	Channel Composition	Testing Conditions
NV1	NP1 + Q1	Visible light (V)
NV2	NP1 + Q2	Visible light (V)
NV3	NP1 + Q3	Visible light (V)
NV4	NP1 + Q4	Visible light (V)
NV5	NP1 + Q5	Visible light (V)
NV6	NP1 + Q6	Visible light (V)
NF1	NP1 + Q1	Fluorescent light (F)
NF2	NP1 + Q2	Fluorescent light (F)
NF3	NP1 + Q3	Fluorescent light (F)
NF4	NP1 + Q4	Fluorescent light (F)
NF5	NP1 + Q5	Fluorescent light (F)
NF6	NP1 + Q6	Fluorescent light (F)
MV1	NP2 + Q1	Visible light (V)
MV2	NP2 + Q2	Visible light (V)
MV3	NP2 + Q3	Visible light (V)
MV4	NP2 + Q4	Visible light (V)
MV5	NP2 + Q5	Visible light (V)
MV6	NP2 + Q6	Visible light (V)
MF1	NP2 + Q1	Fluorescent light (F)
MF2	NP2 + Q2	Fluorescent light (F)
MF3	NP2 + Q3	Fluorescent light (F)
MF4	NP2 + Q4	Fluorescent light (F)
MF5	NP2 + Q5	Fluorescent light (F)
MF6	NP2 + Q6	Fluorescent light (F)

**Table 3:** Key parameters for quantitative analysis of four enantiomers of catechins by PLSR.

Enantiomers	Detection Channel	Detection Range (mol/L)	Coefficient of Determination (R^2^)	Recovery Rate (%)
Catechin	NV2, NV3, NV4, NF2	1 × 10^−7^–1 × 10^−3^	0.99	95.2–103.1
Epicatechin	FV4, NF1	1 × 10^−7^–1 × 10^−3^	0.99	94.8–102.7
Catechin gallate	NV3, NV5	1 × 10^−7^–5 × 10^−4^	0.99	96.5–104.3
Epicatechin gallate	MV5, MF4, MF6	1 × 10^−7^–1 × 10^−3^	0.99	95.7–103.5

**Table 4:** Quantitative results of Longjing tea with the different storage times.

**Label**	**A1**	**A2**	**A3**	**A4**	**A5**	**A6**
R2	0.99	0.99	0.99	0.99	0.99	0.99
Recovery (%)	101.31 ± 0.03	100.81 ± 0.03	98.99 ± 0.02	100.59 ± 0.03	99.44 ± 0.03	100.19 ± 0.03
**Label**	**A7**	**A8**	**A9**	**A10**	**A11**	
R2	0.99	0.99	0.99	0.99	0.99	
Recovery (%)	98.91 ± 0.03	99.56 ± 0.02	100.31 ± 0.03	99.68 ± 0.03	100.49 ± 0.03	

**Table 5:** Sensor comparison table.

Sensor Method	The Distinction of Tea from Different Origins	Authenticity	Enantiomer Mixing Quantification
Fluorescence hyperspectral imaging [48]	×	✓	×
Electrochemical [25]	×	×	×
Indicated displacement array [49]	✓	×	×
This work	✓	✓	✓

## Data Availability

The original contributions presented in the study are included in the article/supplementary material; further inquiries can be directed to the corresponding authors.

## Supplementary Materials

The following supporting information can be downloaded at: https://www.mdpi.com/article/10.3390/foods14193360/s1, Table S1. The detailed information of QDs; Table S2. Linear analysis results table of the Stern-Volmer equation; Table S3. CV-ANOVA for OPLSDA; Table S4. O-PLSDA analysis results of 11 kinds of Xihu Longjing; Table S5. The content of catechin components in 11 kinds of Xihu Longjing by HPLC-MS; Table S6. Quantitative analysis results of catechin components in Xihu Longjing tea with different storage times by HPLC-MS; Figure S1. Morphology and structure characterization of QDs by TEM; Figure S2. The zeta potential results of Q3, Q3 + NP2, and Q3 + NP2+Shifengshan super (**A**–**C**); Figure S3. Fluorescence lifetime of (**A**) Q3; (**B**) Q3 + NP2; (**C**) Q3 + NP2 + A1. (**D**) Photobleaching tests; Figure S4. Fluorescence and UV spectral characterization of NP1 + Q5 after the addition of enantiomers or Longjing tea; Figure S5. Catechin enantiomers; Figure S6. **A**–**D** are, respectively, catechin (5 × 10^−6^–1 × 10^−8^ mol/L), epicatechin (5 × 10^−6^–1 × 10^−8^ mol/L), catechin gallate (9 × 10^−6^–1 × 10^−9^ mol/L), and epicatechin gallate (5 × 10^−6^–1 × 10^−9^ mol/L). E–H are the linear analysis results of the Stern-Volmer equation for catechin, epicatechin, catechin gallate, and epicatechin gallate, respectively; Figure S7. The mixture ratio of catechin gallate and epicatechin gallate is from 1:9 to 9:1; Figure S8. The results of quantitative analysis of the mixture ratio of enantiomers using the PLSR model; Figure S9. The result of enantiomers was quantitatively analyzed by the PLSR model; Figure S10. Xihu Longjing from adjacent origins color discrimination results visualization; Figure S11. Statistical verification for Xihu Longjing classification. (**A**) cross-validation accuracy; (**B**) permutation test results (n = 999); Figure S12. External blind validation of Xihu Longjing classification; Figures S13–16. Color difference diagram of different storage times; Figure S17. The color difference in black tea; Figure S18. Prediction results by OCPLS for other batches of adulterated samples (1:001) and black tea samples; Figure S19. (**A**) Color difference and real photos of tea brewing at different temperatures; (**B**) Ascorbic acid effects on the color difference and the real photo; Figure S20. The color difference map of the visual sensor array with different proportions of tea adulteration; Figure S21. The color difference in the stability and anti-interference test of sensor arrays; Figure S22. Color difference in the sensor during intra-day (morning, noon, and evening) and inter-day (1, 3, and 7 days).
