# Supplementary material for: Catechin-Targeted Nano-Enhanced Colorimetric Sensor Array Based on Quantum Dots—Nano Porphyrin for Precise Analysis of Xihu Longjing from Adjacent Origins
Source: Foods. 2025 Sep 28;14(19):3360. doi: 10.3390/foods14193360 (PMC12523400; doi:10.3390/foods14193360)
Supplement: Supplementary file 1 [file foods-14-03360-s001.zip › foods-3849477-supplementary.pdf]

Table S1.The detail information of QDs

Number	Label	Name	Colour	λ emmax
**1**	Q1	NAC50'@CdTe	Red	650nm
**2**	Q2	GSH@CdTe	Orange	575nm
**3**	Q3	NAC40'@CdTe	Orange	580nm
**4**	Q4	MSA@CdTe	Green	535nm
**5**	Q5	MSA:NAC3:1@CdT	Green	550nm
**6**	Q6	MSA:NAC1:1@CdT	Green	525nm

Table S2. Linear analysis results table of the Stern-Volmer equation

Catechin enantiomers	K_{SV}（L/mol）	Detection channel
catechin	0.08	MF3
epicatechin	0.09	MF3
catechin gallate	0.26	MF5
epicatechin gallate	0.22	NF1

Table S3. CV – ANOVA for OPLSDA

M1	SS	DF	MS	F	p	SD
**Total corr.**	650	650	1			1
**Regression**	646.623	280	2.30937	252.998	0	1.51966
**Residual**	3.37736	370	0.009128			0.0955406

Table S4.O-PLSDA analysis results of 11 kinds of Xihu Longjing

	R2X	R2X(CUM)	Elgenvalue	R2	R2(CUM)	Q2	Limit	Q2(CUM)	R2Y	R2Y(CUM)	ElgenvalueY	Significance
Modle			0.99		0.998			0.994		1		
Predictive			0.525		0.998			0.994		1		
P1	0.248	0.248	16.4	0.1	0.1	0.0882	0.01	0.0882	0.1	0.1	1.1	R1
P2	0.0866	0.334	5.72	0.0997	0.2	0.0893	0.01	0.178	0.1	0.2	1.1	R1
P3	0.06	0.403	4.52	0.09	0.3	0.08	0.0	0.266	0.1	0.3	1.1	R1

	48			99		85	1					
P4	0.0401	0.443	2.64	0.1	0.4	0.0979	0.01	0.364	0.1	0.4	1.1	R1
P5	0.029	0.472	1.91	0.1	0.5	0.105	0.01	0.469	0.1	0.5	1.1	R1
P6	0.0188	0.491	1.24	0.0999	0.6	0.1	0.01	0.569	0.1	0.6	1.1	R1
P7	0.0125	0.503	0.826	0.1	0.699	0.106	0.01	0.676	0.1	0.7	1.1	R1
P8	0.008	0.512	0.582	0.0999	0.799	0.0974	0.01	0.773	0.1	0.8	1.1	R1
P9	0.008	0.52	0.559	0.0995	0.899	0.113	0.01	0.886	0.1	0.9	1.1	R1
P10	0.004	0.525	0.298	0.0993	0.998	0.108	0.01	0.994	0.1	1	1.1	R1
Orthogonal in X(OPLS)		0.465			0							
O1	0.194	0.194	12.8	0	0							R1
O2	0.0996	0.294	6.57	0	0							R1
O3	0.0863	0.38	5.7	0	0							R1
O4	0.0367	0.417	2.42	0	0							R1
O5	0.0268	0.444	1.77	0	0							R1
O6	0.0128	0.456	0.848	0	0							R1
O7	0.008	0.465	0.576	0	0							R1

Table S5. The content of catechin components in 11 kinds of Xihu Longjing by HPLC-MS

Sample	Catechin content（mg/g）	Epicatechin content（mg/g）	Catechin gallate Content（mg/g）	Epicatechin gallate content（mg/g）
A1	1.688	6.547	33.125	0.981
A2	1.364	6.913	30.974	0.636
A3	1.136	5.278	32.577	0.944

A4	1.277	6.423	32.676	1.129
A5	1.496	7.542	33.36	0.898
A6	1.46	7.922	31.22	0.761
A7	1.185	6.761	27.23	0.537
A8	0.884	4.697	28.308	0.574
A9	1.748	6.849	37.268	0.95
A10	1.413	5.638	31.429	0.813
A11	1.262	5.558	31.169	0.892

Table S6. Quantitative analysis results of catechin components in Xihu Longjing tea with different storage times by HPLC-MS

	**Catechin（μg/g）**	**Epicatechin（μg/g）**	**Catechin gallate（μg/g）**	**Epicatechin gallate（μg/g）**
**Meijiawu-First- Day 1**	1.43	6.61	32.11	1.21
**Meijiawu-First- Day 5**	1.19	6.63	32.59	1.12
**Meijiawu-First- Day 20**	1.62	7.44	33.38	0.94
**Meijiawu-First- Day 60**	1.86	7.64	25.5	0.93

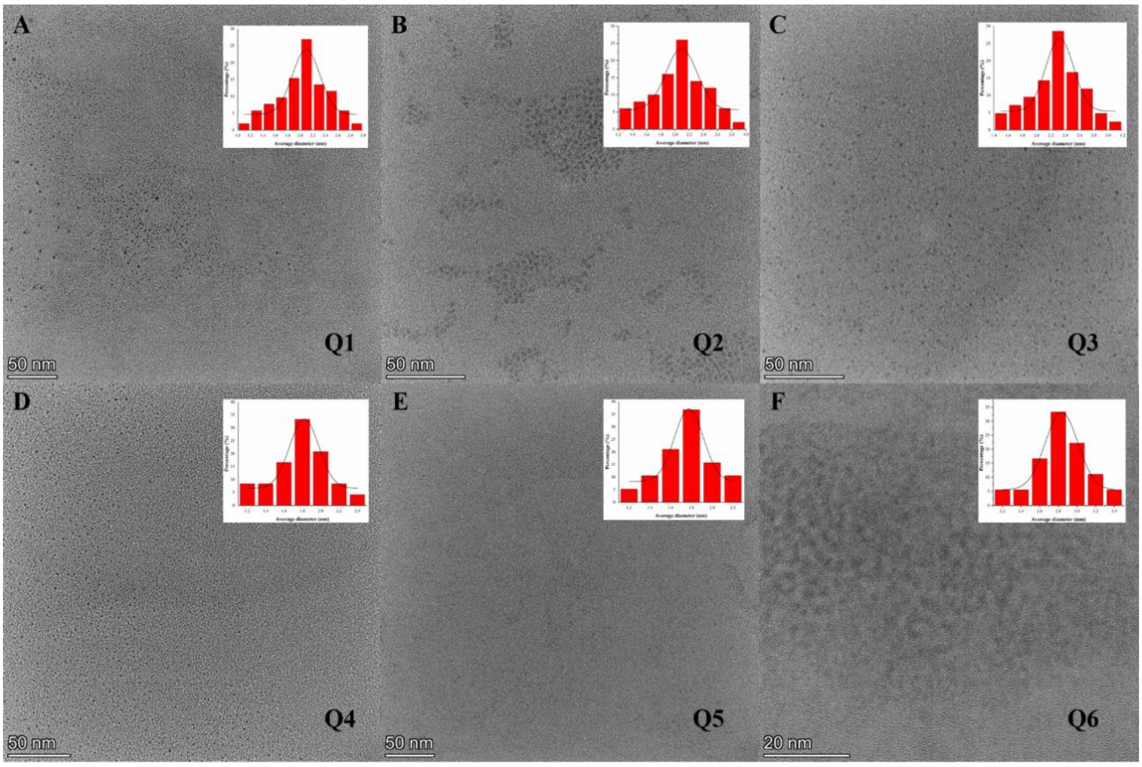

Figure S1.   Morphology and structure characterization of QDs by TEM

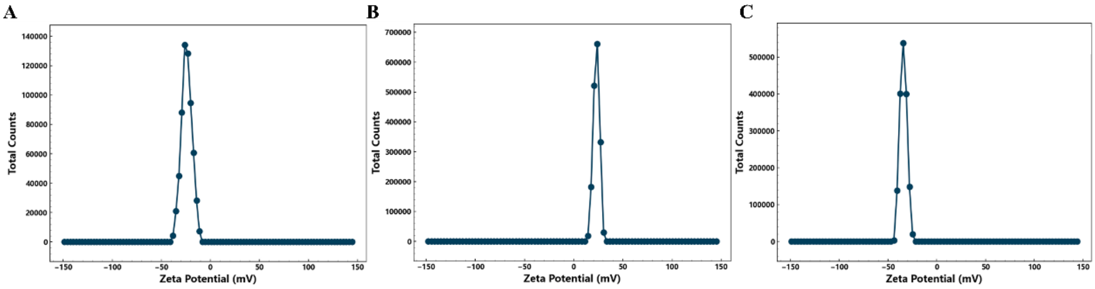

Figure S2. The zeta potential results of Q3, Q3+NP2, and Q3+NP2+Shifengshan super (A-C)

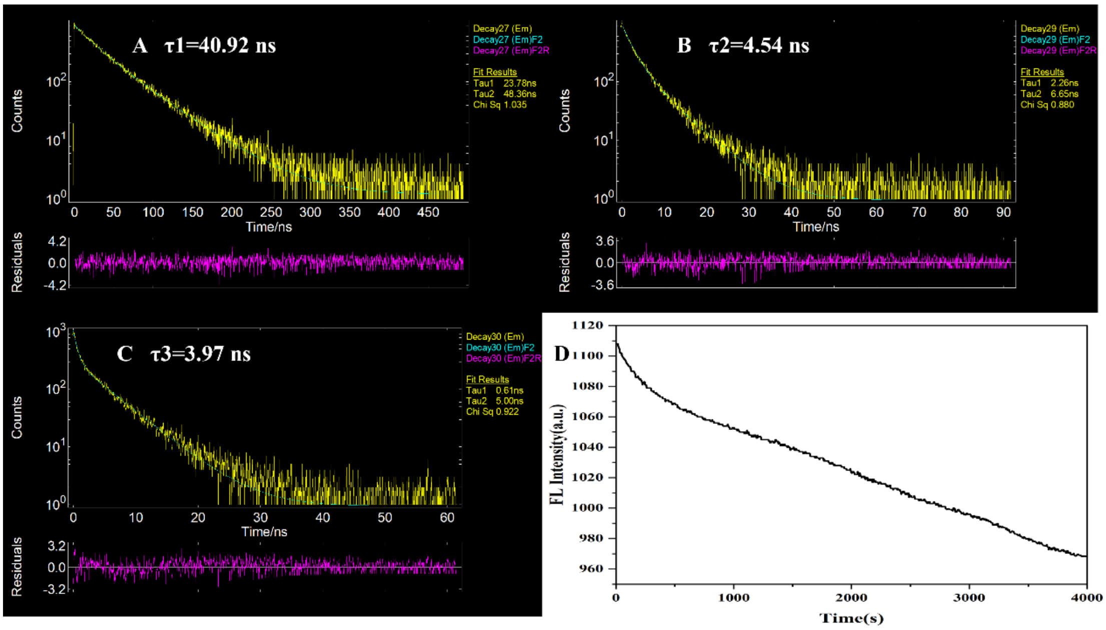

Figure S3. Fluorescence lifetime of (A) Q3; (B) Q3+NP2; (C) Q3+NP2+A1. (D) Photobleaching tests.

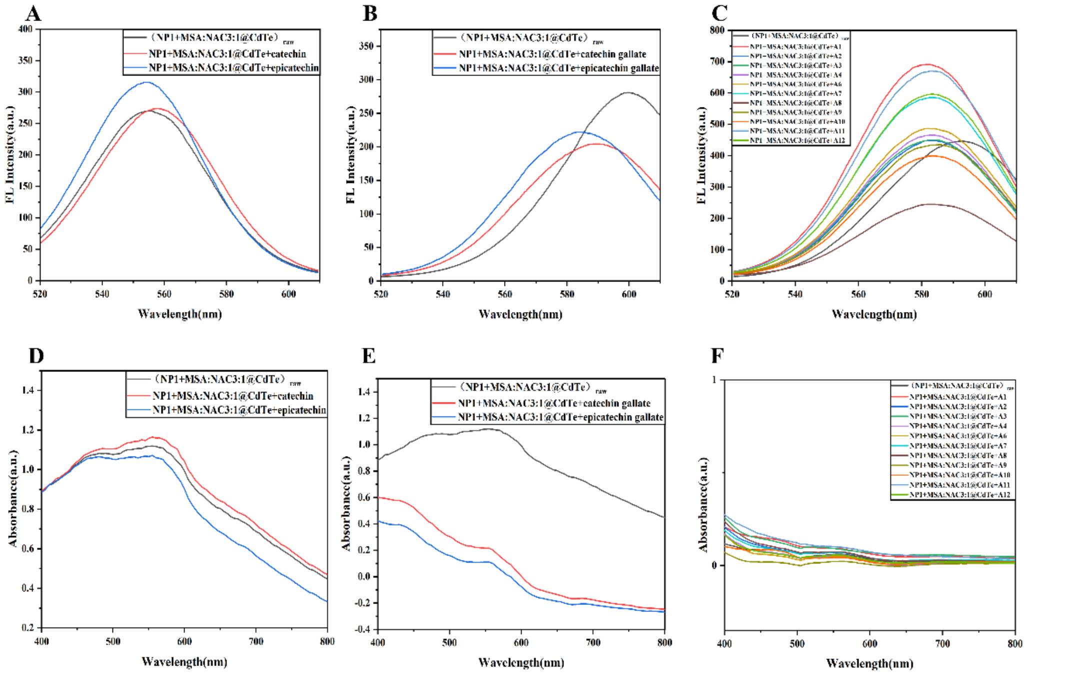

Figure S4. Fluorescence and UV spectral characterization of NP1+Q5 after the addtion of enantiomers or Longjing tea. (A) Fluorescence spectral response of NP1+Q5 to catechins and epicatechin (1×10^{-5} mol/L). (B) Fluorescence spectral response of NP1+Q5 to catechin gallate and epicatechin gallate (1×10^{-5} mol/L). (C) Fluorescence spectral response of NP1+Q5 to Longjing teas. (D) UV spectral response of NP1+Q5 to catechins and epicatechin (1×10^{-5} mol/L). (E) UV spectral response of NP1+Q5 to catechin gallate and epicatechin gallate (1×10^{-5} mol/L). (F) UV spectral response of NP1+Q5 to Longjing teas.

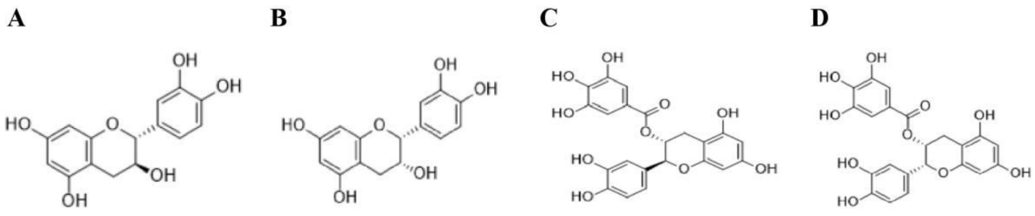

Catechin **Epicatechin** **Catechin gallate** **Epicatechin gallate**

Figure S5. Catechin enantiomers. (A) Catechin (B) Epicatechin (C) Catechin gallate (D) Epicatechin gallate

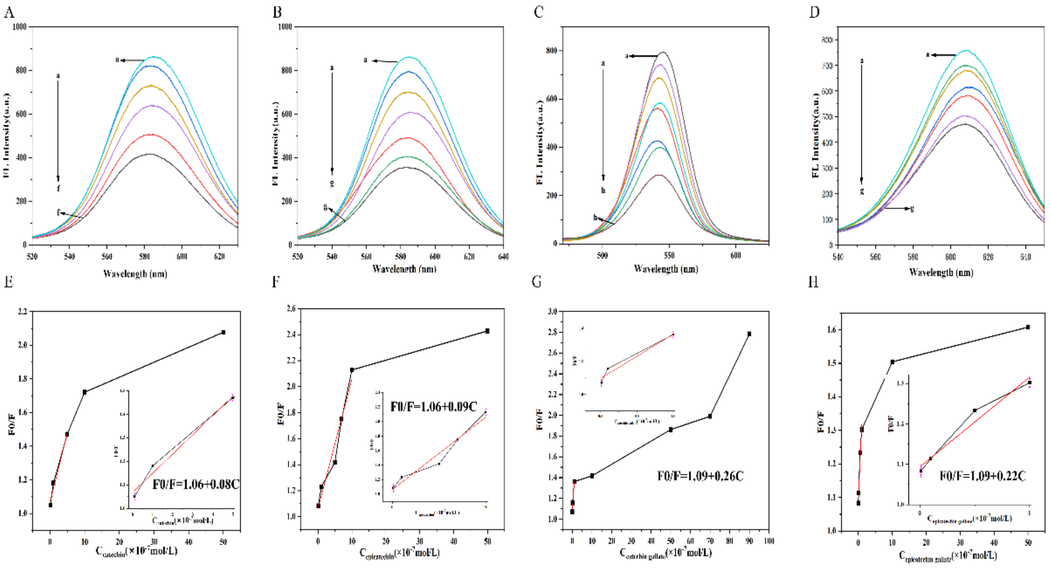

Figure S6. A-D are respectively catechin (5×10^{-6}-1×10^{-8}mol/L), epicatechin (5×10^{-6}-1×10^{-8}mol/L), catechin gallate (9×10^{-6}-1×10^{-9}mol/L) and epicatechin gallate (5×10^{-6}-1×10^{-9}mol/L). E-H are the linear analysis results of the Stern-Volmer equation for catechin, epicatechin, catechin gallate, and epicatechin gallate, respectively.

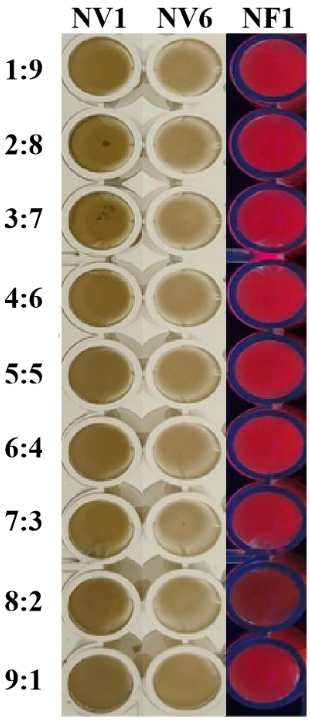

Figure S7. The mixture ratio of catechin gallate and epicatechin gallate from 1:9 to 9:1

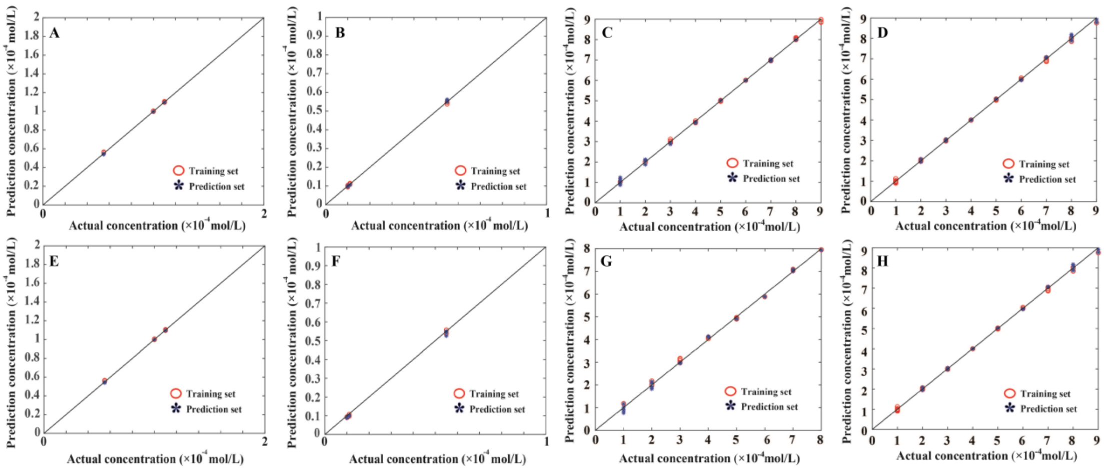

Figure S8. The results of quantitative analysis of the mixture ratio of enantiomers using PLSR model: the mixture ratio of catechin and epicatechin from 1:1 to 1:0.01 (A, B); the mixture ratio of catechin and epicatechin from 1:9 to 9:1 (C, D). The mixture ratio of catechin gallate and epicatechin gallate from 1:1 to 1:0.01 (E, F); the mixture ratio of catechin gallate and epicatechin gallate from 1:9 to 9:1 (G, H).

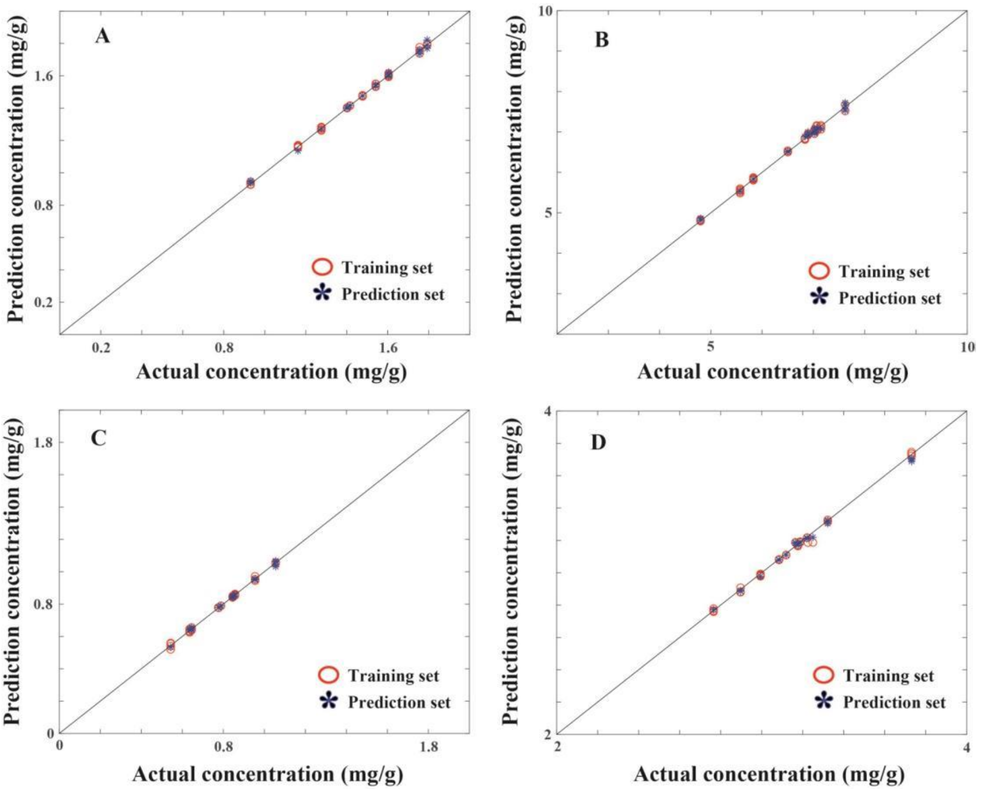

Figure S9. The result of enantiomers was quantitatively analyzed by PLSR model

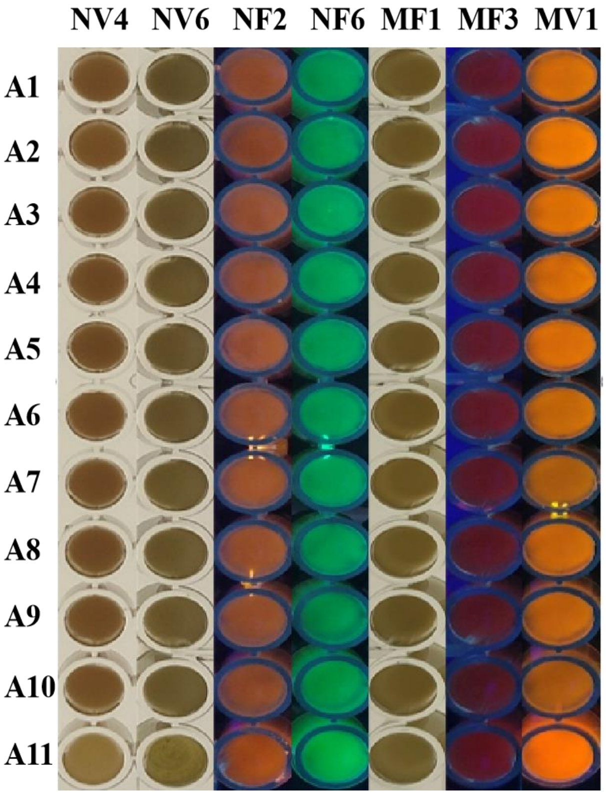

Figure S10. Xihu Longjing from adjacent origins color discrimination results visualization

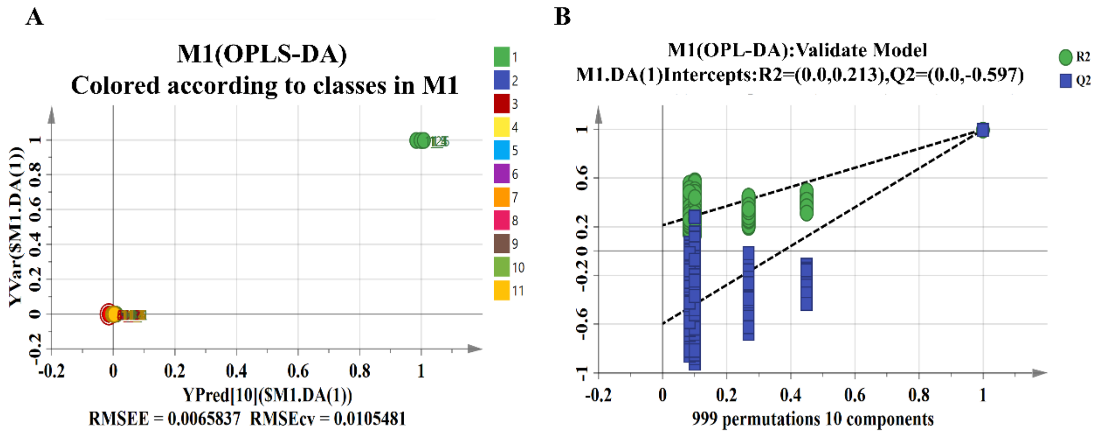

Figure S11. Statistical verification for Xihu Longjing classification. (A) cross-validation accuracy; (B) permutation test results (n=999)

`

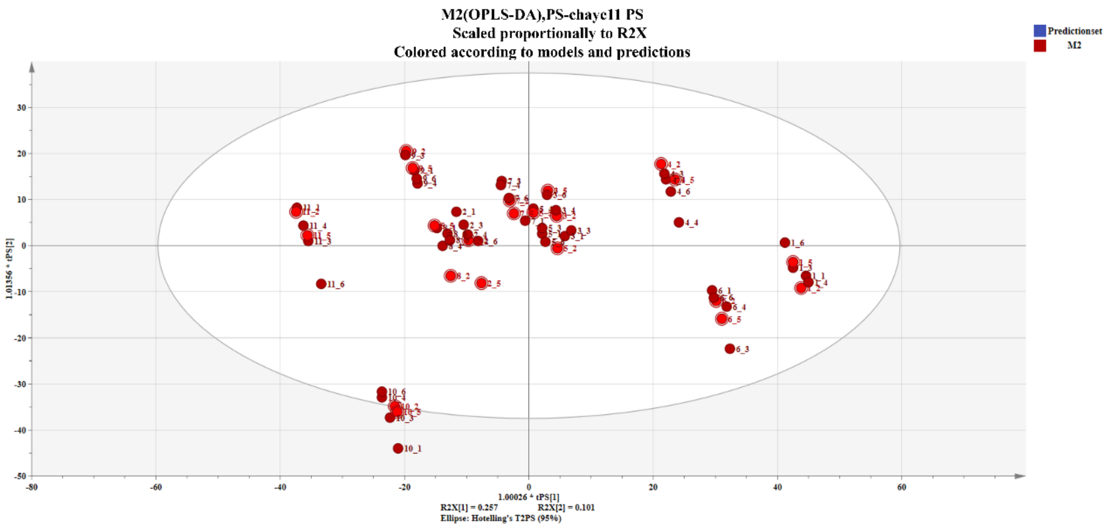

Figure S12. External blind validation of Xihu Longjing classification

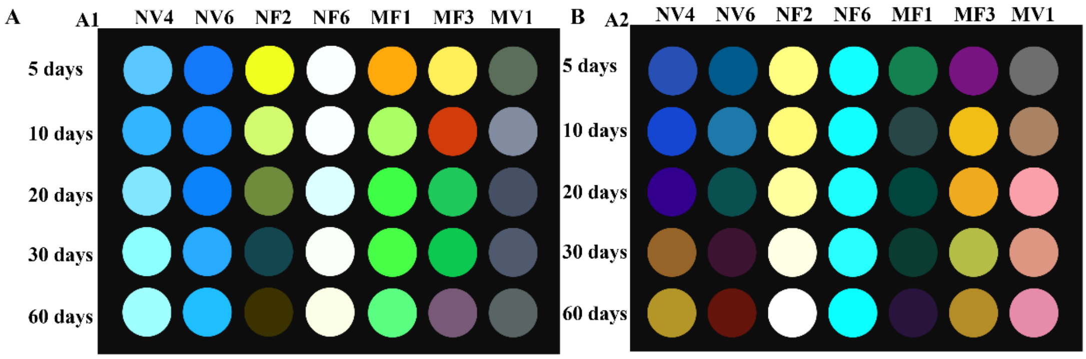

Figure S13. Color difference diagram of different storage time

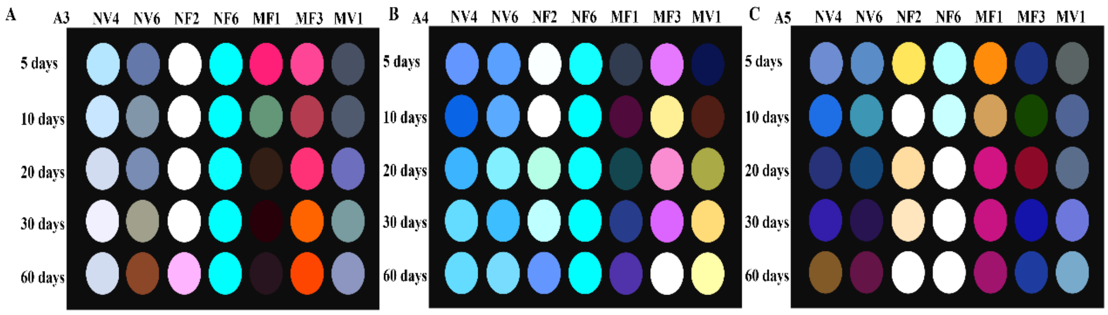

Figure S14. Color difference diagram of different storage time

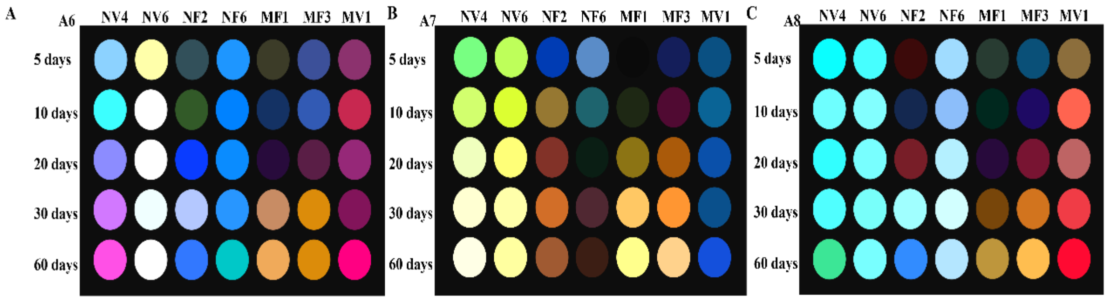

Figure S15. Color difference diagram of different storage time

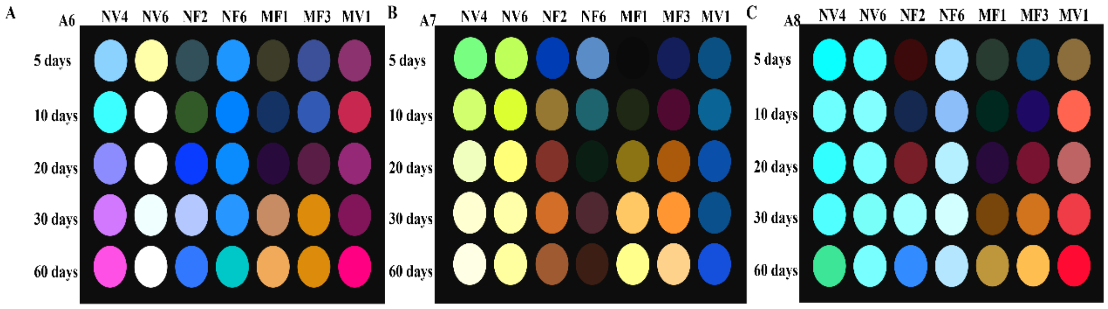

Figure S16. Color difference diagram of different storage time

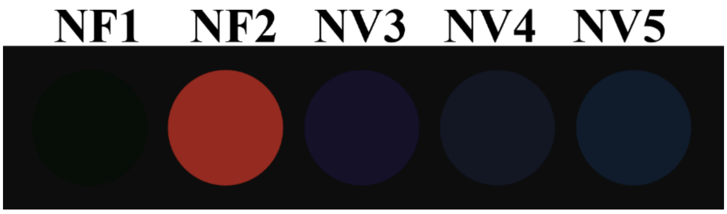

Figure S17. The color difference of black tea

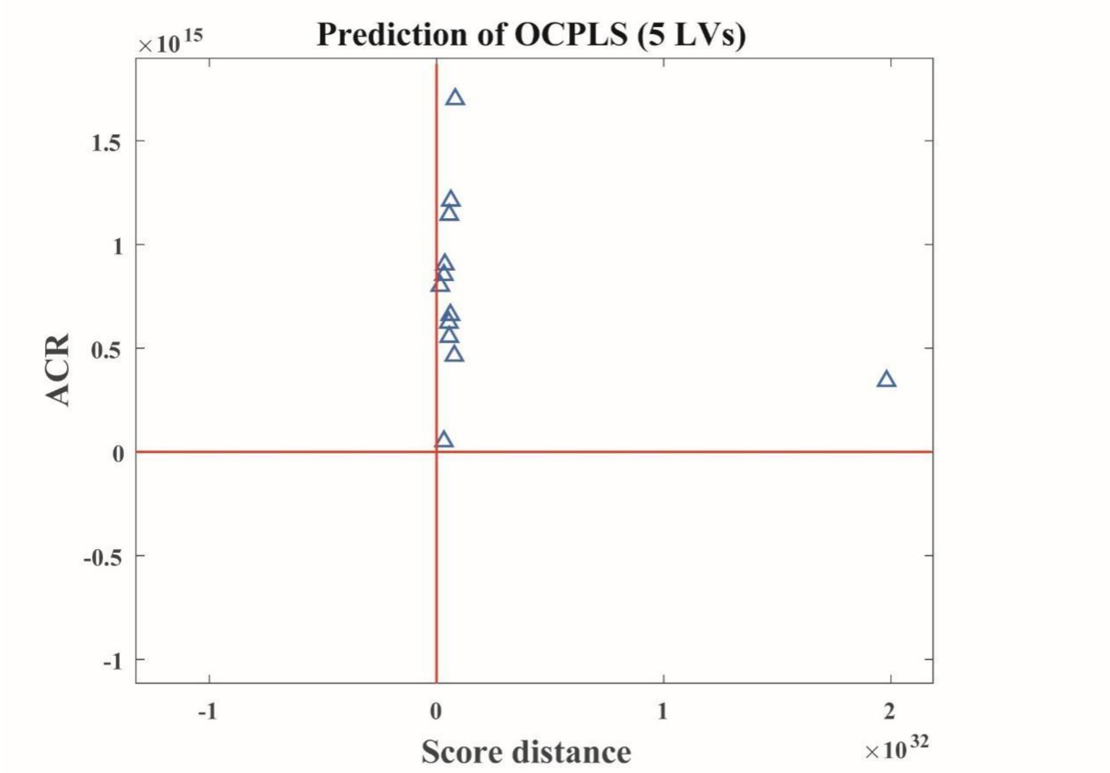

Figure S18. Prediction results by OCPLS for other batches of adulterated samples (1:001) and black tea samples

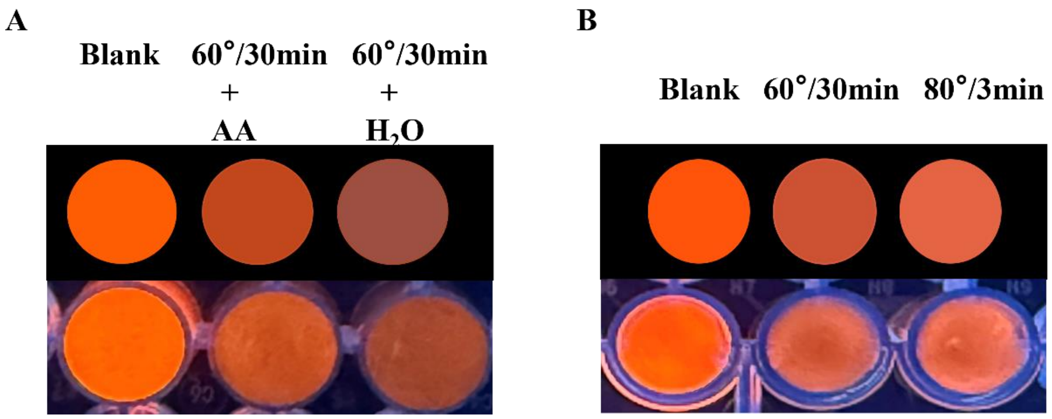

Figure S19. (A) Color difference and real photos of tea brewing at different temperatures; (B) Ascorbic acid effects on the color difference and the real photo.

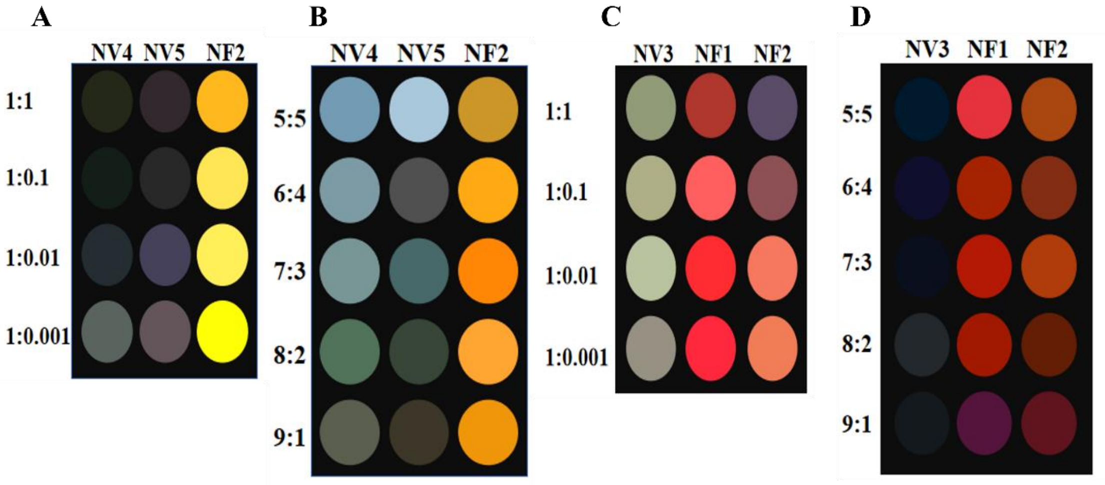

Figure S20. The color difference map of visual sensor array with different proportions of tea adulteration: (A) Longjing teas from Shifengshan mixed with Longjing teas from Meijiawu with the ratio ranging from 1:1-1:0.001; (B) Longjing teas from Shifengshan mixed with Longjing teas from Meijiawu with the ratio ranging from 9:1-5:5. (C) Longjing teas from Shifengshan mixed with Wuniuzao with the ratio ranging from 1:1-1:0.001; (D) Longjing teas from Shifengshan mixed with Wuniuzao with the ratio ranging from 9:1-5:5.

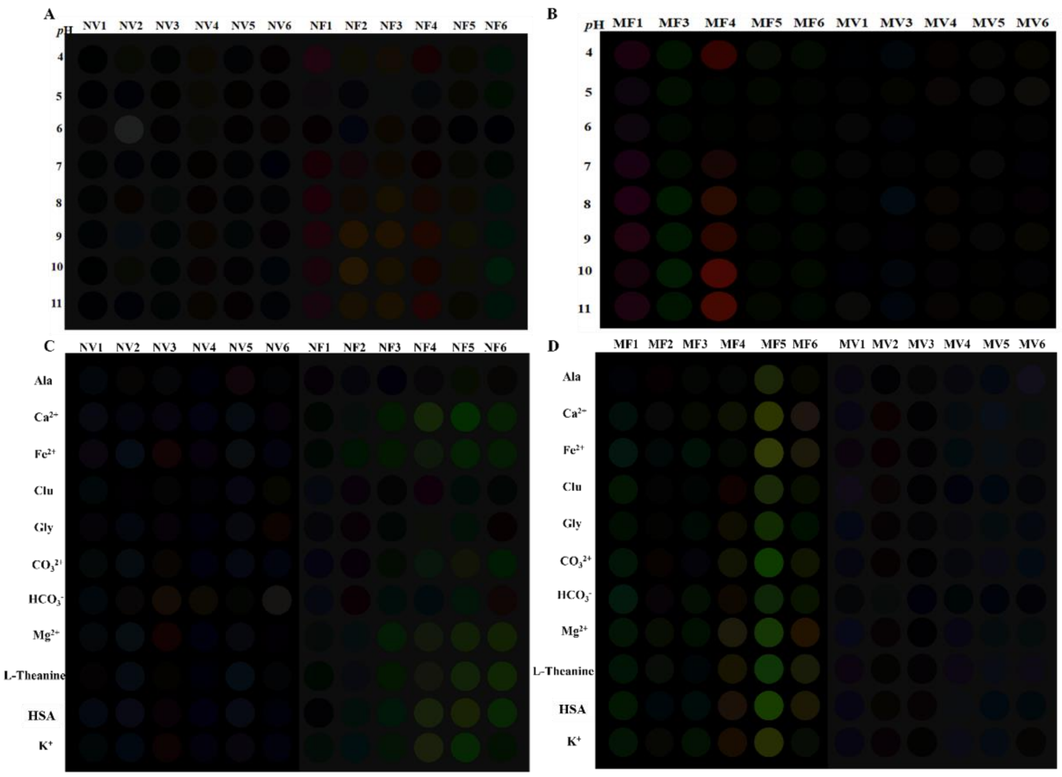

Figure S21. The color difference of stability and anti-interference test of sensor arrays.

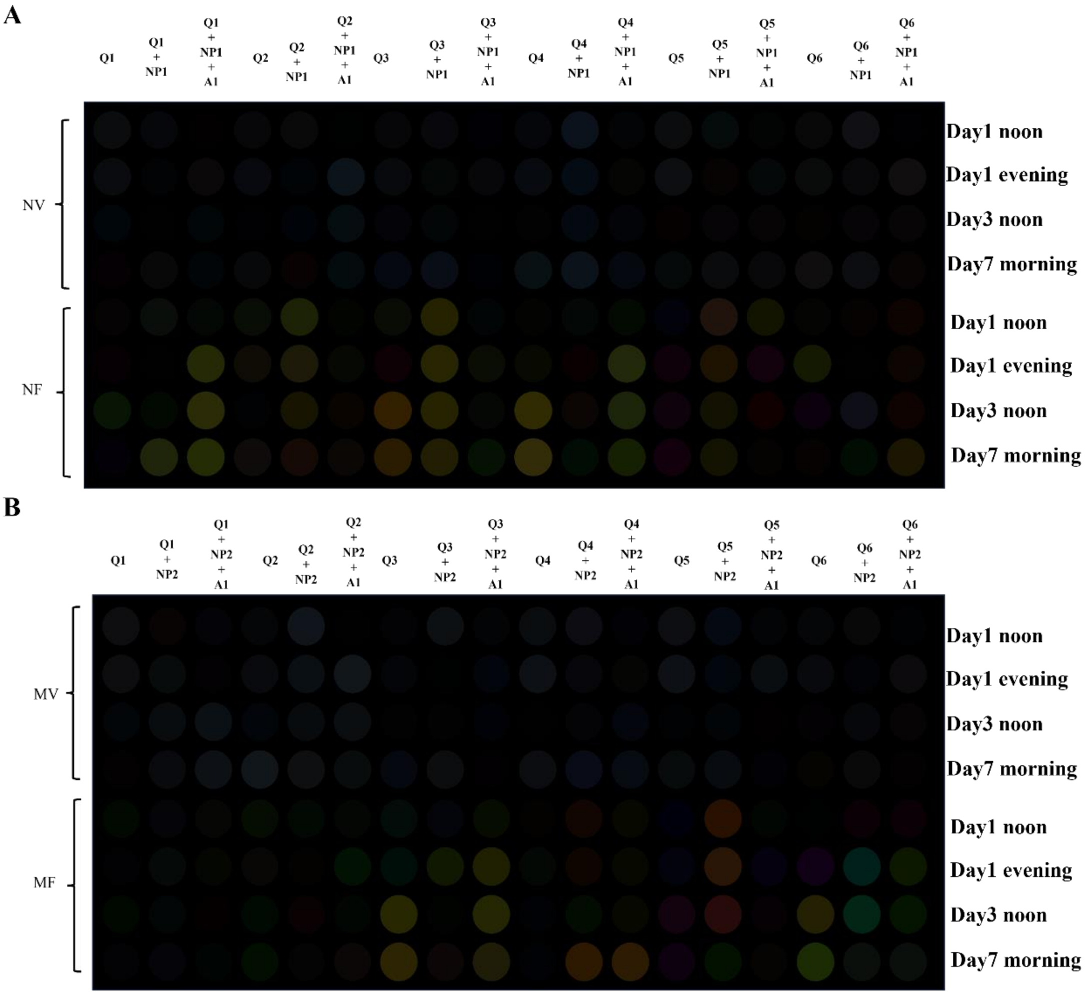

Figure S22. Color difference of the sensor during intra-day (morning, noon, evening) and inter-day (1, 3, 7 days)
